# Cytosporones with Anti-Inflammatory Activities from the Mangrove Endophytic Fungus *Phomopsis* sp. QYM-13

**DOI:** 10.3390/md21120631

**Published:** 2023-12-07

**Authors:** Guisheng Wang, Yilin Yuan, Zhaokun Li, Junhao Zhu, Zhigang She, Yan Chen

**Affiliations:** 1 School of Pharmacy, Anhui Medical University, Hefei 230032, China; wanggsh9@mail2.sysu.edu.cn (G.W.); lizhaokun0223@163.com (Z.L.); 19855683468@163.com (J.Z.); 2 School of Chemistry, Sun Yat-sen University, Guangzhou 510275, China; 3 National R&D Center for Edible Fungus Processing Technology, Henan University, Kaifeng 475004, China; yilin@henu.edu.cn

**Keywords:** mangrove endophytic fungus, *Phomopsis* sp., cytosporone, anti-inflammatory

## Abstract

Six previously undescribed cytosporone derivatives (phomotones A-E (**1**–**5**) and phomotone F (**13**)), two new spiro-alkanol phombistenes A-B (**14**–**15**), and seven known analogs (**6**–**12**) were isolated from the mangrove endophytic fungus *Phomopsis* sp. QYM-13. The structures of these compounds were elucidated using spectroscopic data analysis, electronic circular dichroism (ECD), and ^13^C NMR calculations. Compound **14** features an unprecedented 1,6-dioxaspiro[4.5]decane ring system. All isolates were evaluated for their inhibitory effect on nitric oxide (NO) in LPS-induced RAW264.7 cells. The results showed that compounds **1**, **6**, **8,** and **11** exhibited potent bioactivities by comparing with positive control. Then, compound **1** displayed the anti-inflammatory effect by inhibiting the MAPK/NF-*κ*B signaling pathways. Molecular docking further revealed the possible mechanism of compound **1** interaction with ERK protein.

## 1. Introduction

Cytosporones are a series of polyketide-derived octaketide phenolic lipids [1], the first metabolite of which is cytosporone A, isolated from an endophytic fungi *Cytospora* sp. in 2000 [2]. These compounds were likewise obtained from other fungi, like *Dothiorella*, *Pestalotiopsis*, *Diaporthe*, *Phomopsis*, *Aspergillus*, *Trichoderma*, and so on [3]. Furthermore, cytosporones were reported to have various kinds of biological activities, such as antimicrobial, antimalarial, cytotoxic, antivirus, anti-inflammatory, and allelopathic activity [4,5,6].

**Figure 1:**
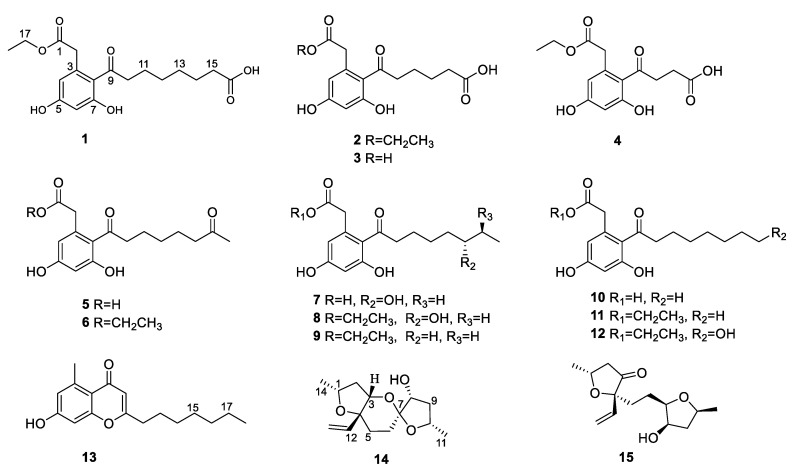
The structures of **1**–**15**.

Mangrove endophytic fungi have attracted the attention of many researchers due to their ability to produce structurally novel and remarkably bioactive secondary metabolites [7]. To date, thousands of new metabolites have been isolated from mangrove endophytic fungi [8]. As part of our ongoing search for bioactive compounds from mangrove endophytic fungi, the strain *Phomopsis* sp. QYM-13, isolated from healthy leaves of *Kandelia candel*, was investigated. We recently reported twelve new cytochalasins, phomopchalasins D–O, including brominated and iodinated cytochalasins, with significant cytotoxicity from this fungus [9]. Subsequently, another eight new cytosporones phomotones A-E (**1**–**5**), phomotone F (**13**), and phombistenes A-B (**14**–**15**) (Figure 1) were obtained in our further research work. In bioassays, compounds **1**, **6**, **8,** and **11** exhibited significant anti-inflammatory activities. Herein, the isolation, structure elucidation, biological assays, molecular docking, and structure-activity relationship of these isolated compounds are described.

## 2. Results

**Figure 2:**
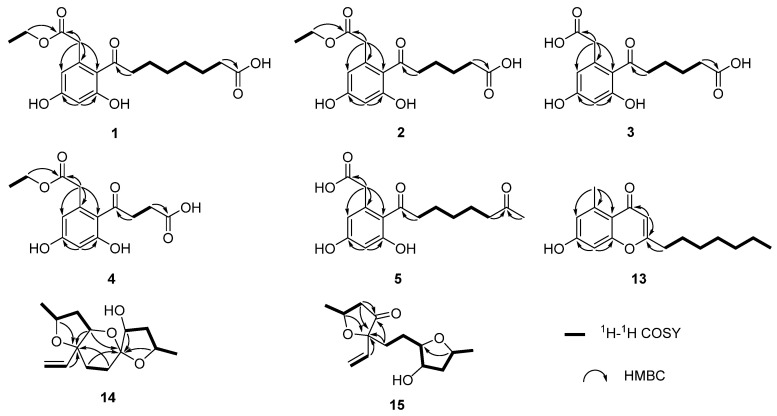
The key ^1^H-^1^H COSY and HMBC correlations of compounds **1**–**5** and **13**–**15**.

Compound **1** was isolated as a white solid with the molecular formula of C_18_H_23_O_7_ based on the negative HRESIMS data *m*/*z* 351.14534 [M‒H]^−^. The ^1^H NMR spectrum of **1** showed the presence of two aromatic protons (*δ*_H_ 6.20 (d, *J* = 2.2 Hz), 6.26 (d, *J* = 2.2 Hz)) and one methyl group (*δ*_H_ 1.24 (t, *J* = 7.1 Hz)). The ^13^C NMR (Table 1) and HSQC spectra (Figure S3) of **1** revealed the coexistence of eighteen carbons attributable to one methyl, eight methylenes (one oxygenated), two methines, and seven unprotonated carbons (three carbonyl carbons and four olefinic carbons). These data suggested **1** to be a cytosporone class, and the ^1^H and ^13^C NMR data of **1** were similar to those of **12 [10]**. The main difference involved the absence of the oxygenated methylene in **12** and the appearance of carboxyl carbon in **1**, which might mean the terminal hydroxyl group in **12** was oxidized to a carboxyl group in **1**. The deduction was further confirmed with the spin system of H_2_-10/H_2_-11/H_2_-12/H_2_-13/H_2_-14/H_2_-15 in the ^1^H-^1^H COSY spectrum (Figure 2), as well as the HMBC correlations (Figure 2) from H_2_-10 to C-9 and from H_2_-15 to C-16. Thus, the structure of **1** was assigned as shown in Figure 1.

Compound **2** was isolated as a colorless oil with the molecular formula of C_16_H_19_O_7_ based on the negative HRESIMS data *m*/*z* 323.11392 [M‒H]^−^. Detailed analysis of the NMR data (Table 1) indicated that **2** was structurally similar to **1**, with the difference being the absence of two methylenes. It was supported by its ^1^H-^1^H COSY cross-peaks of H_2_-10/H_2_-11/H_2_-12/H_2_-13 and HMBC correlations from H_2_-10 to C-9 and from H_2_-13 to C-14. Thus, the structure of **2** was established.

Compound **3** was isolated as a colorless oil with the molecular formula of C_14_H_15_O_7_ based on the negative HRESIMS data *m*/*z* 295.08227 [M‒H]^−^. The ^1^H and ^13^C NMR data of **3** (Table 1) were similar to those of **2**, except for the absence of an ethyl group (*δ*_C_ 60.4, 13.1) in **3**. Then, the HMBC correlations (Figure 2) further confirmed the deduction above.

Compound **4** was isolated as a colorless oil with the molecular formula of C_14_H_15_O_7_ based on the negative HRESIMS data *m*/*z* 295.08240 [M‒H]^−^. A comparison of the NMR data of **4** (Table 2) with those of **2** revealed that their structures were similar, with the only difference being the absence of two methylenes. The ^1^H-^1^H COSY cross-peaks of H_2_-10/H_2_-11 and HMBC correlations from H_2_-10 to C-9 and from H_2_-11 to C-12 further confirmed the structure.

Compound **5** was isolated as a colorless oil with the molecular formula of C_16_H_21_O_6_ based on the positive HRESIMS data *m*/*z* 309.1333 [M + H]^+^. The ^1^H and ^13^C NMR data of **5** (Table 2) were similar to those of **6 [10]**, except for the absence of an ethyl group (*δ*_C_ 61.5, 14.2) in **5**. The deduction was further confirmed by ^1^H-^1^H COSY and HMBC correlations (Figure 2).

For compound **13**, a yellow solid, the molecular formula C_17_H_22_O_3_ with seven degrees of unsaturation was established using HRESIMS. The NMR (Table 2) information of **13** was similar to **10**, with the main difference being the presence of one methyl group (*δ*_H_ 2.64), one olefin proton (*δ*_H_ 5.95), and two olefin carbons (*δ*_C_ 167.3, 110.5) in **13**, and the disappearance of the carboxyl group at C-1 in **10**. The HMBC correlations from H_3_-2 to C-3, C-4, and C-8, from H-10 to C-9, C-11, and C-12, together with the spin system of H_2_-12/H_2_-13/H_2_-14/H_2_-15/H_2_-16/H_2_-17/H_3_-18 in the ^1^H-^1^H COSY spectrum (Figure 2) further confirmed the deduction. Thus, the structure of **13** was verified.

For compound **14**, a yellow solid, the molecular formula C_14_H_22_O_4_ with four degrees of unsaturation was established using HRESIMS. The ^1^H NMR spectrum (Table 3) showed two methyl groups at *δ*_H_ 1.34 (d, *J* = 6.2 Hz) and 1.30 (d, *J* = 6.4 Hz), and three olefin protons at *δ*_H_ 5.73 (ddd, *J* = 4.1, 10.7, 14.7 Hz), *δ*_H_ 5.07 (d, *J* = 10.7 Hz), and *δ*_H_ 5.30 (d, *J* = 17.1 Hz). The ^13^C NMR and HSQC spectra of **14** revealed the existence of fourteen carbons attributable to two methyls, five methylenes (one olefinic carbon), five methines (one olefinic carbon), and two unprotonated carbons. From an analysis of the ^1^H-^1^H COSY spectrum, four independent coupling fragments could be inferred (Figure 2). Furthermore, the HMBC correlations from H-1, H-3, H-6, and H2-13 to C-4 and from H-3 and H-5 to C-7 indicated the furan ring and pyranoid ring were fused at positions C-3/C-4, and one group of terminal olefin was located at C-4. Then, the HMBC correlations from H-8 and H-10 to C-7 and the chemical shift at C-4 (*δ*_C_ 108.5) enabled the construction of a 1,6-dioxaspiro[4.5]decane ring system. Therefore, the planar structure of **14** was tentatively assigned.

**Figure 3:**
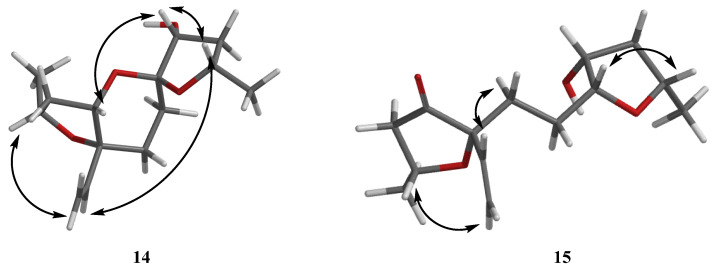
NOESY correlations of **14**–**15**.

**Figure 4:**
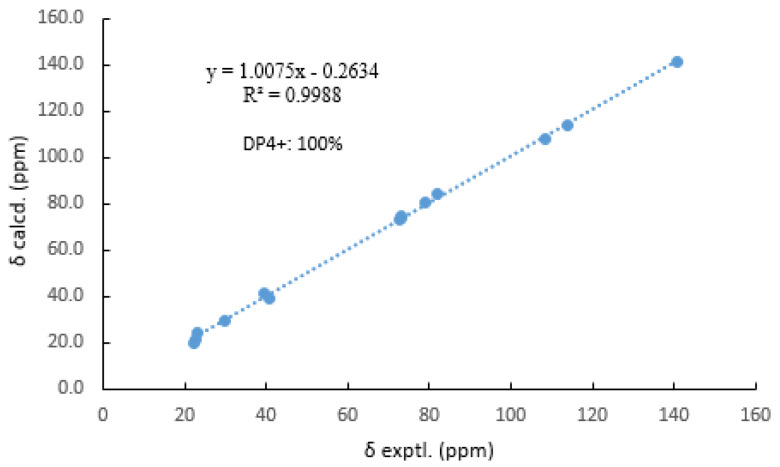
Comparisons of calculated and experimental ^13^C NMR data of **14**.

**Figure 5:**
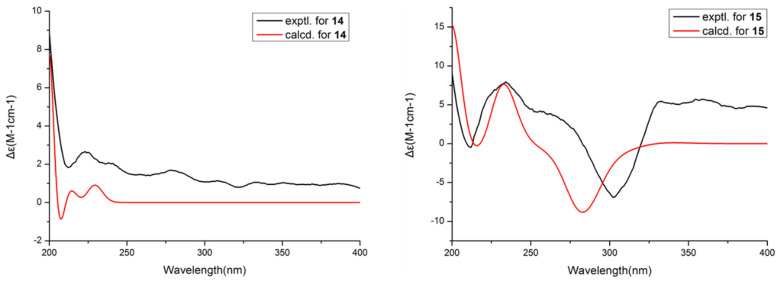
Experimental and calculated ECD spectra of **14** and **15**.

Then, the relative configuration of **14** was determined through analysis of its ^1^H NMR and NOESY correlations (Figure 3). The cross-peaks of H-3/H-8/H-10 and H-1/H_2_-13/H-10 suggested that these protons were co-facial. However, the configuration at C-7 was difficult to determine due to the absence of a correlation between H_2_-6 and H-8. Subsequently, the ^13^C NMR calculations of (1R*, 3S*, 4R*, 7S*, 8R*, 10S*)-**14a** and (1R*, 3S*, 4R*, 7R*, 8R*, 10S*)-**14b** were carried out using the GIAO method at mPW1PW91-SCRF/6-311+G (d, p)/PCM (MeOH). The results showed that **14a** was the most likely candidate structure, with a better correlation coefficient (R^2^ = 0.9988) and a high DP4+ probability of 100% (all data) probability (Figure 4). Thereafter, the ECD calculated was performed at the rB3LYP/6-311G level to determine the absolute configuration of **14**. The calculated curve matched well with its experimental ECD curve (Figure 5). Thus, the absolute configuration of **14** was assigned as 1*R*, 3*S*, 4*R*, 7*S*, 8*R*, 10*S*.

For compound **15**, a yellow solid, its molecular formula was determined as C_14_H_22_O_4_ using HRESIMS data. The NMR (Table 3) data of **15** were similar to those of **14**, indicating that the structure of **15** closely resembled **14**. The main difference was that the oxygenated methine at C-3 in **14** was oxidated to a carbonyl group in **15**. Then, the spin systems of H_3_-14/H-1/H_2_-2 and H_2_-5/H_2_-6/H-7/H-8/H_2_-9/H-10/H_3_-11 from the ^1^H-^1^H COSY spectrum (Figure 2) and the HMBC correlations (Figure 2) from H-2 and H-5 to C-3 and C-4, and from H-10 to C-7, supported that the oxygen bridge bond between C-3 and C-7 was split. With the consideration of biogenetic origin, and the NOESY correlations (Figure 3) of H-1/H_2_-13 and H-8/H-10, the relative configuration of **15** was established as 1R*, 4R*, 7R*, 8R*, and 10S*. Then, the similarity of experimental and calculated ECD curves (Figure 5) allowed the assignment of the absolute configuration of **15** as 1*R*, 4*R*, 7*R*, 8*R*, and 10*S*.

The other compounds were identified as dothiorelone I (**6**) [10], (*S*)-dothiorelone Q (**7**) [11], dithiorelone B (**8**) [12], dithiorelone A (**9**) [13], cytoporone A (**10**) [1], cytoporone B (**11**) [1], a dothiorelone J (**12**) [10] by comparing the spectroscopic data to the literature.

Numerous inflammatory targets including iNOS, COX-1, COX-2, ICAM, IL-5, IL-17, JAK1, JAK2, SIRT2, and TNF-α were investigated through virtual screening for all compounds. The results indicated that iNOS showed stronger binding affinity with **1**–**13** than other targets (Table S2). Thereafter, all compounds were evaluated for their inhibitory activities against LPS-induced nitric oxide (NO) production in RAW 264.7 mouse macrophages. At non-cytotoxic concentrations, the results showed (Table 4) that compounds **1**, **6**, **8,** and **11** exhibited significant anti-inflammatory activities with IC_50_ values of 10.0, 12.0, 13.4, and 11.5 μM, respectively. Compounds **2**, **12,** and **13** showed potent anti-inflammatory activities compared with the positive control (_L_-NMMA: 32.8 μM). Thereafter, the preliminary structure–activity relationship was discussed. The compounds **2**, **6**, **8**, and **11** displayed higher anti-inflammatory activity than **3**, **5**, **7,** and **10**, which indicated that the acetyl group at C-**1** is beneficial for activity. The carboxyl group in the side chain at C-16 may contribute to the activity by comparing **1** with **6**, **8**, **9**, **11,** and **12**. Moreover, the number of carbons in the side chain at C-8 may has not affected the anti-inflammatory activity by comparing the IC_50_ values of **1**–**12**.

**Figure 6:**
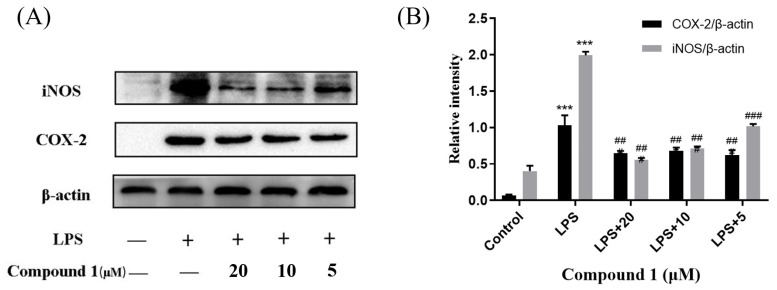
Influences of compound **1** on iNOS, COX-2, and β-actin protein expression were detected using western blotting (**A**); the ratio of the content of iNOS/β-actin and COX-2/β-actin (**B**). Data rendered are the mean ± SD, *n* = 3. In comparison to the control, *** *p* < 0.001. In comparison to the LPS group, ### *p* < 0.001, ## *p* < 0.01.

Cytosporones were reported to have anti-inflammatory activity. To explore the anti-inflammatory mechanism of these compounds, the inhibitory effects of inflammation-related iNOS and COX-2 for new compound **1** were measured using western blot. As a result, the protein expression of iNOS and COX-2 were apparently down-regulated after treatment of **1** with different concentrations (20.0, 10.0, and 5.0 μM) in a dose-dependent manner and dose-independent manner, respectively (Figure 6). These results were consistent with the project of target docking.

**Figure 7:**
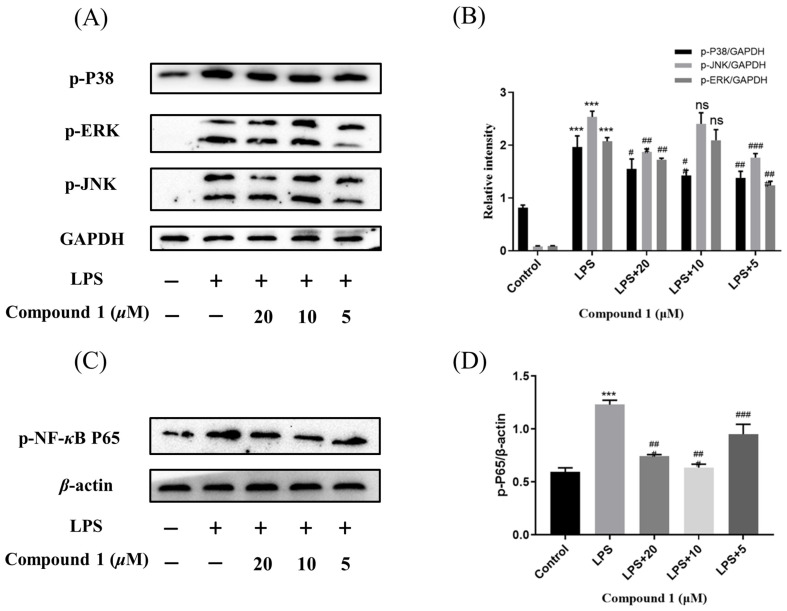
Influences of compound **1** on the MAPK and NF-*κ*B pathway detected with western blotting. (**A**) The expression levels of p-JNK, p-ERK, p-P38, and GAPDH detected with Western blotting. (**B**) The proportion of p-JNK, p-ERK, and p-P38 to GAPDH content. (**C**) The expression levels of p-P65 and β-actin detected with western blotting. (**D**) The proportion of p-P65 to β-actin content. Data rendered are the mean ± SD, *n* = 3. In comparison to the control, *** *p* < 0.001. In comparison to the LPS, # *p* < 0.05, ## *p* < 0.01, ### *p* < 0.001.

Mitogen-activated protein kinases (MAPK) and nuclear factor-κB (NF-κB) pathway were known to regulate the inflammatory response by modulating multiple pro-inflammatory cytokines in macrophages. So as to elucidate the mechanism of action by which compound **1** inhibited the level of NO, the MAPK and NF-*κ*B signaling pathway of **1** was further investigated. As expected, the phosphorylation levels of p38, JNK, and ERK were significantly up-regulated after the treatment of RAW264.7 cells with LPS in the MAPK signaling pathway. Meanwhile, the phosphorylation levels of p38, JNK, and ERK were reduced after pretreatment of RAW264.7 cells with different concentrations of compound **1**. In addition, the western blotting methods have detected the expression of phosphorylation p65 in the NF-*κ*B signaling pathway. The results implied that pretreatment with compound **1** obviously decreased the levels of phosphorylation p65 (Figure 7). Taken together, compound **1** displayed the anti-inflammatory effect by inhibiting the MAPK/NF-*κ*B signaling pathways.

**Figure 8:**
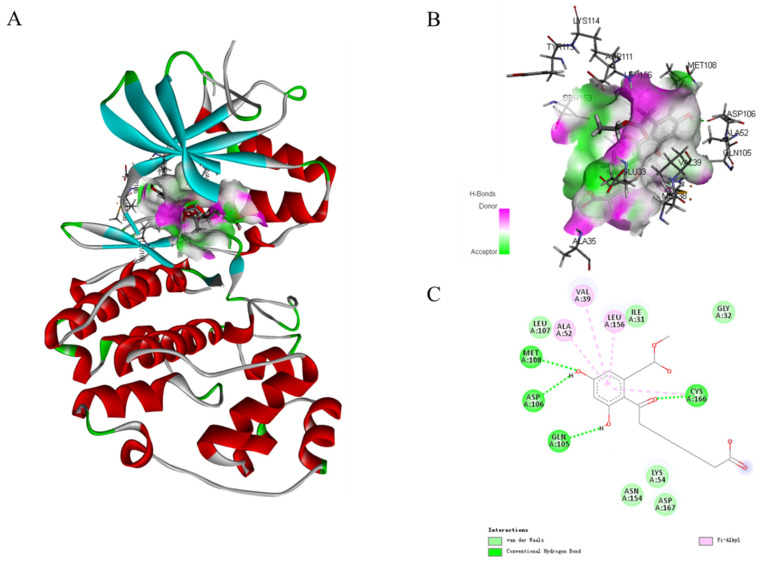
Molecular docking models for ERK (PDB:5v60) inhibition of compound **1** (**A**). Hydrogen bonding active pocket of compound **1** (**B**). 2D interaction diagrams of compound **1** (**C**).

In the MAPK signaling pathway, the levels of phosphorylation ERK were remarkably diminished compared with p38 and JNK. To further expound the role of phosphorylation ERK in compound **1** effects, molecular docking analysis was performed to investigate an inside interaction between compound **1** and ERK protein (PDB:5v60). As shown in Figure 8, the results indicated that **1** binds deeply in the active site pocket between Lys114, Tyr113, Asp111, Ser153, Glu33, Met38, and Ala35. Four hydrogen bonds are formed between the hydroxyl group at C-5 and C-7, and carbonyl at C-9 of **1** with Met108, Asp106, Gln105, and Cys166, respectively. In addition, multiple van der Waals-interacting residues such as Leu107, Ile31, Asn154, Lys54, Asp167, and Gly32 are generated between the ligand and the receptor protein, as well as π–π stacking interaction with Ala52, Val39, and Leu156. Thus, compound **1** could effectively activate the ERK signaling.

## 3. Experimental Section

### 3.1. General Experimental Procedures

The HRESIMS data of compounds **1**–**4**, **5,** and **13**–**15** were obtained using LTQ Orbitrap LC-MS (Thermo Fisher Scientific, Bremen, Germany), Agilent G6230 Q-TOF LC/MS(Agilent, Santa Clara, CA, USA), and a Waters SYNAPT G2-Si (Waters Aisa Ltd., Singapore) mass spectrometer, respectively. All semi-preparative HPLC was performed on an Agilent 1260 liquid chromatography system(Agilent, Santa Clara, CA, USA) with an XSelect CSH C18 column (5 μm, 4.6 × 250 mm). The other procedures were the same as previously reported [9].

### 3.2. Fungal Material, Fermentation and Isolation

The strain *Phomopsis* sp. QYM-13 was described as previously reported [9]. Briefly, the mycelia of the fungus were inoculated into a 250 mL potato dextrose medium for 5 days to prepare the seed culture. Thereafter, the spore suspension was transferred into liquid mediums each containing 300 mL (3 g of potato extract, glucose 20 g/L, artificial sea salts 20 g/L, 100 × 1 L Erlenmeyer flasks) for 30 days at 25 °C. Then, the extracts (21.2 g) were obtained from the mycelia and broth as the methods previously described. It was subjected to silica gel column chromatography (CC, 200 mesh silica) eluting with petroleum ether/EtOAc (9:1 to 1:9) to yield 6 fractions (Fr. A-F). Fr. A was fractionated with Sephadex LH-20 CC (CH_2_Cl_2_/MeOH *v*/*v*, 1:1) to yield 2 fractions (Fr. A1-A2). Fr. A1 was further separated using silica gel CC (CH_2_Cl_2_/MeOH *v*/*v*, 97:3) and HPLC eluting with MeOH/H_2_O (*v*/*v*, 86:14) to afford compounds **3** (3.4 mg) and **5** (4.6 mg). Fr. A2 was isolated using silica gel CC (CH_2_Cl_2_/MeOH *v*/*v*, 95:5) and further purified by semipreparative HPLC (MeOH-H_2_O, 79:21) to give compounds **1** (2.2 mg) and **13** (6.7 mg). Fr. C was purified on Sephadex LH-20 CC (100% MeOH) to provide fractions C1-C3. Compound **2** (4.3 mg) and compound **14** (3.3 mg) were obtained from Fr. C3, which were purified using HPLC (MeOH-H_2_O, 77:23). Fr. D was subjected to Sephadex LH-20 CC (CH_2_Cl_2_/MeOH *v*/*v*, 1:1) to give fractions D1-D2. Fr. D1 was isolated using silica gel CC (CH_2_Cl_2_/MeOH *v*/*v*, 93:7) to yield compounds **6** (8.3 mg) and **4** (5.6 mg). Fr. D2 was purified using HPLC (MeOH-H_2_O, 75:25) to give compounds **8** (3.2 mg) and **9** (2.8 mg). Fr. E was subjected to silica gel CC (CH_2_Cl_2_/MeOH *v*/*v*, 85:15) to give fractions E1-E3. Fr. E2 was purified using semipreparative HPLC (MeOH-H_2_O, 73:27) to give compounds **7** (1.9 mg), **10** (3.5 mg), and **15** (2.8 mg). Fr. E3 was purified using HPLC eluting with MeOH-H_2_O (*v*/*v*, 70:30) to give compounds **11** (2.6 mg) and **12** (3.3 mg).

Compound **1**: white solid, UV (MeOH) λ_max_ (logε): 220 (3.10), 232 (3.23), 271 (2.90), 288 (3.35) nm. IR (KBr): ν_max_ 3310, 1728, 1600, 1471, 1358, 1231 cm^−1^. ^1^H and ^13^C NMR (500 MHz, MeOD-d_4_) data, Table 1. HRESIMS *m*/*z* 351.14534 [M‒H]^−^ (calcd for C_18_H_23_O_7_, 351.14531).

Compound **2**: colorless oil, UV (MeOH) λ_max_ (logε): 218 (3.15), 235 (3.20), 263 (2.89), 290 (3.41) nm. IR (KBr): ν_max_ 3286, 1726, 1610, 1465, 1235 cm^−1^. ^1^H and ^13^C NMR (500 MHz, MeOD-d_4_) data, see Table 1. HRESIMS *m*/*z* 323.11392 [M‒H]^−^ (calcd for C_16_H_19_O_7_, 323.11390).

Compound **3**: colorless oil, UV (MeOH) λ_max_ (logε): 226 (3.08), 242 (2.80), 271 (3.0), 301 (3.38) nm. IR (KBr): ν_max_ 3310, 1727, 1600, 1463, 1362, 1230 cm^−1^. ^1^H and ^13^C NMR (500 MHz, MeOD-d_4_) data, see Table 1. HRESIMS *m*/*z* 295.08227 [M‒H]^−^ (calcd for C_14_H_15_O_7_, 295.08229).

Compound **4**: colorless oil, UV (MeOH) λ_max_ (logε): 218 (3.0), 236 (3.06), 272 (2.85), 293 (3.23) nm. IR (KBr): ν_max_ 3286, 1731, 1605, 1469, 1370, 1231 cm^−1^. ^1^H and ^13^C NMR (500 MHz, MeOD-d_4_) data, see Table 2. HRESIMS *m*/*z* 295.08240 [M‒H]^−^ (calcd for C_14_H_15_O_7_, 295.08242).

Compound **5**: colorless oil, UV (MeOH) λ_max_ (logε): 217 (3.10), 226 (2.90), 270 (2.89), 291 (3.32) nm. IR (KBr): ν_max_ 3288, 1731, 1600, 1463, 1365, 1230 cm^−1^. ^1^H and ^13^C NMR (500 MHz, MeOD-d_4_) data, see Table 2. HRESIMS *m*/*z* 309.1333 [M + H]^+^ (calcd for C_16_H_21_O_6_, 309.1328).

Compound **13**: yellow solid, UV (MeOH) λ_max_ (log ε) 228 (1.55) nm. IR (KBr) υ_max_ 3435, 2970, 1717 cm^−1^. ^1^H and ^13^C NMR (500 MHz, DMSO-d_6_) data, see Table 2. HRESIMS *m*/*z* 275.6262 [M + H]^+^ (calcd for C_17_H_23_O_3_, 275.6263).

Compound **14**: yellow solid, [α${]}_{D}^{25}$ = +8.5 (c 0.33, CH_3_OH). UV (MeOH) λ_max_ (log ε) 228 (1.55) nm. IR (KBr) υ_max_ 3435, 2970, 1717 cm^−1^. ^1^H and ^13^C NMR (CDCl_3_) data, see Table 3. HRESIMS *m*/*z* 253.1436 [M‒H]^−^ (calcd for C_14_H_21_O_4_, 253.1434).

Compound **15**: yellow solid, [α${]}_{D}^{25}$ = +7.5 (c 0.33, CH_3_OH). UV (MeOH) λ_max_ (log ε) 224 (2.01) nm. IR (KBr) υ_max_ 3430, 2973, 1675 cm^−1^. ^1^H and ^13^C NMR (CDCl_3_) data, see Table 3. HRESIMS *m*/*z* 255.1587 [M + H]^+^ (calcd for C_14_H_23_O_4_, 255.1589).

### 3.3. ECD and NMR Calculation Methods

The ECD calculation was performed as described previously [7,14]. The conformers were subjected to geometric optimization at the level of B3LYP/6-31+G in the liquid phase. Thereafter, the optimized conformers were calculated using the TD-DFT method at the RB3LYP/6-311G (**14**) and PBEPBE/LAN12DZ (**15**) levels, respectively.

Typically, the Merck molecular force field in Spartan’s 10 software was used for the conformational analysis of compound **14**. Conformers with populations exceeding 5% according to the Boltzmann distribution were optimized using the B3LYP/6-311+G (d, p) level in the polarizable continuum model (PCM) with methanol as the solvent. Subsequently, NMR calculations were performed using the gauge invariant atomic orbital (GIAO) method at the mPW1PW91-SCRF/6-311+G (d, p) level with PCM in methanol (Gaussian 09). Finally, the shielding constants were averaged using Boltzmann distribution theory for each stereoisomer, and their experimental and calculated data were analyzed using DP4+ probability.

### 3.4. Anti-inflammatory Assay

Cell culture: the RAW264.7 cells were cultured in Dulbecco’s modified Eagle’s medium (DMEM, Gibco, Grand Island, NY, USA) containing 10% fetal bovine serum (FBS, Grand Island, NY, USA) and 1% double antibodies (100 U/mL penicillin and 100 μg/mL streptomycin) at 37 °C with a 5% CO_2_ humidified incubator.

Cell viability assay: the cell viability was measured using an MTT assay into 96-well plates. Approximately 3 × 10^6^ cells/mL were inoculated overnight at 37 °C with 5% CO_2_. Then, the cells were pretreated with different concentrations of L-NMMA or compound (5, 10, 20, 30, 40, and 50 μM) for 24 h. Thereafter, approximately 10 μL of MTT (0.5 mg/mL) was added to each well for 4 h. The absorbance was checked at 540 nm.

Bioassay of NO production: cells were inoculated overnight in 24-well plates with a density of 3 × 10^6^ cells/mL (500 μL/well). Various concentrations of compounds were pretreated with LPS (1 μg/mL) for 24 h. The content of NO was measured according to the instructions of the Griess assay. The absorbance of the final product was measured at 540 nm.

Western blotting: cells (1 × 10^6^ cells/well) were inoculated in 6-well plates with DMEM. Then, the cells were pretreated with compound **1** (20, 10, and 5 μM) and incubated for 24 h. The detailed operation process was according to the methods described previously [15].

### 3.5. Molecular Docking Studies

The molecular docking screening was performed using Sybyl-X 2.0 [16]. The three-dimensional structure of protein (iNOS: 2ORP; COX-1: 1HT8; COX-2: 5F19; ICAM: 6S8T; IL-17: 4HSA; IL-5: 3QT2; JAK1: 6N7A; JAK2: 6X8E; SIRT2: 4RMH; TNF-α: 2AZ5; p-ERK: 5V60) was obtained from the RCSB Protein Data Bank. The method used for the molecular docking screening was previously reported [17]. Briefly, the receptor compounds **1**–**13** were first optimized with the Gaussian View 5 program at DFT calculations. Then, the ligand substructures were extracted, and water molecules were removed. Subsequently, the target compounds were docked into the pocket of a receptor using Sybyl-X 2.0.

## 4. Conclusions

Six new cytosporone derivatives (phomotones A-D (**1**–**5**) and phomotone F (**13**)), two new spiro-alkanol phombistenes A-B (**14**–**15**), and seven known analogs (**6**–**12**) were isolated from the mangrove endophytic fungus *Phomopsis* sp. QYM-13. All compounds were evaluated for their inhibitory activities against LPS-induced nitric oxide (NO) production in RAW 264.7 macrophages after virtual screening using molecular docking with numerous inflammatory targets. The results showed that compounds **1**, **6**, **8,** and **11** exhibited promising anti-inflammatory activities. Thereafter, the mechanism of action suggested that compound **1** displayed the anti-inflammatory effect by inhibiting the MAPK/NF-κB signaling pathways. Furthermore, compound **1** could effectively activate the ERK signaling through hydrogen bonds, van der Waals, and π–π stacking. This research indicated that the cytosporone derivatives could be developed as anti-inflammatory therapeutic lead compounds.

## Figures and Tables

**Table 1:** ^1^H (500 MHz) and ^13^C (125 MHz) NMR data for compounds **1**–**3** in MeOD-*d*_4_.

	1		2		3	
No	*δ*_C_	*δ*_H_ (*J* in Hz)	*δ*_C_	*δ*_H_ (*J* in Hz)	*δ*_C_	*δ*_H_ (*J* in Hz)
1	173.6		172.1		173.9	
2	40.5	3.58, s	39.0	3.60, s	38.9	3.57, s
3	137.0		135.7		135.8	
4	111.7	6.20, d (2.2)	110.4	6.21, d (2.2)	110.2	6.21, d (2.2)
5	161.3		159.9		160.2	
6	102.7	6.26, d (2.2)	101.3	6.28, d (2.2)	101.3	6.26, d (2.2)
7	159.8		158.4		157.0	
8	121.3		119.8		120.3	
9	208.9		206.9		207.1	
10	45.1	2.91, t (7.4)	43.3	2.96, t (7.0)	43.2	2.95, m
11	25.4	1.62, m	23.6	1.68, m	23.6	1.65, m
12	30.1	1.35, m	24.5	1.65, m	24.4	1.62, m
13	30.1	1.33, m	33.6	2.32, t (7.0)	33.4	2.30, m
14	26.0	1.59, m	176.3		175.6	
15	35.1	2.28, d (7.4)	60.4	4.12, q (7.1)		
16	178.0		13.1	1.26, t (7.1)		
17	61.8	4.11, q (7.1)				
18	14.5	1.24, d (7.1)				

**Table 2:** ^1^H (500 MHz) and ^13^C (125 MHz) NMR data for compounds **4–5** and **13**.

	4*^a^*		5*^a^*		13*^b^*	
No	*δ*_C_	*δ*_H_ (*J* in Hz)	*δ*_C_	*δ*_H_ (*J* in Hz)	*δ*_C_	*δ*_H_ (*J* in Hz)
1	172.3		176.2			
2	39.2	3.58, s	41.0	3.58, s	22.8	2.64, s
3	139.0		137.9		141.9	
4	110.5	6.19, d (2.2)	111.6	6.23, d (2.2)	116.9	6.60, d (2.2)
5	160.1		161.4		159.6	
6	101.5	6.27, d (2.2)	102.6	6.26, d (2.2)	101.0	6.62, d (2.2)
7	158.8		159.8		161.3	
8	119.3		121.3		114.8	
9	204.5		209.5		178.7	
10	38.7	3.20, t (6.8)	25.1	1.64, m	110.5	5.95, s
11	28.5	2.60, t (6.8)	35.3	1.30, m	167.3	
12	175.8		29.7	1.34, m	33.1	2.53, t (7.0)
13	60.4	4.11, q (7.1)	24.7	1.57, m	26.6	1.61, m
14	13.1	1.24, t (7.1)	44.2	2.49, t (7.4)	28.7	1.32, m
15			212.3	2.14, s	28.7	1.30, m
16			29.8		22.5	1.28, m
17					31.6	1.24, m
18					14.4	0.85, t (6.8)

*^a^* Measured in MeOD-*d*_4_, *^b^* measured in DMSO-*d*_6_.

**Table 3:** ^1^H (500 MHz) and ^13^C (125 MHz) NMR data for compounds **14–15** in CDCl_3_.

	14		15	
No	*δ*_C_	*δ*_H_ (*J* in Hz)	*δ*_C_	*δ*_H_ (*J* in Hz)
1	73.0	4.33, dt (6.2,19.6)	70.2	4.43, ddd (3.3, 6.1,12.2)
2a	40.7	2.52, m	40.8	1.66, dd (2.9, 9.0)
2b		1.44, dd (4.8,13.6)		1.55, dd (2.5, 5.6)
3	72.9	4.24, d (5.8)	214.9	
4	81.8		86.4	
5	23.1	1.85, m	30.8	1.78, m
6	29.8	1.94, m	27.2	1.59, dd (5.0, 9.0)
7	108.5		74.0	3.71, ddd (3.1, 5.8, 9.0)
8	79.0		72.0	3.46, m
9a	39.4	2.21, ddd (5.9, 7.8, 13.5)	43.1	2.58, dd (6.1, 17.8)
9b		1.72, dd (3.8, 5.0)		2.27, dd (9.4, 17.8)
10	73.2	4.14, td (6.9, 12.6)	65.6	4.14, ddd (3.1, 6.3, 8.8)
11	22.4	1.34, d (6.2)	23.7	1.24, d (6.3)
12	140.7	5.73, ddd, (4.1, 10.7, 14.7)	137.1	5.78, dd (10.7,17.1)
13a	113.8	5.30, d (17.1)	116.2	5.45, dd (1.4,17.1)
13b		5.07, d (10.7)		5.23, dd (1.4,10.7)
14	22.6	1.30, d (6.4)	22.2	1.44, d (6.09)

**Table 4:** The anti-inflammatory activities of compounds **1**–**15**.

Comp.	1	2	3	4	5	6	7	8
IC_50_ (μM)	10.0 ± 0.3	17.2 ± 1.0	38.6 ± 0.3	42.1 ± 1.5	>50	12.0 ± 0.5	>50	13.4 ± 0.5
Comp.	**9**	**10**	**11**	**12**	**13**	**14**	**15**	_L_-NMMA ^a^
IC_50_ (μM)	>50	47.0 ± 2.3	11.5 ± 0.3	28.2 ± 1.0	25.0 ± 1.2	25.6 ± 0.8	30.2 ± 0.3	32.8 ± 0.2

^a^ positive control.

## Data Availability

The data presented in this study are available in Supplementary Materials.

## Supplementary Materials

The following supporting information can be downloaded at https://www.mdpi.com/article/10.3390/md21120631/s1, HRESIMS, 1D and 2D NMR spectra of compounds **1**–**5** and **13**–**15**, the DP4+ evaluation of compound **14**, and the binding energy of compounds **1**–**13** with numerous inflammatory targets.
