# Supplementary material for: Cytosporones with Anti-Inflammatory Activities from the Mangrove Endophytic Fungus Phomopsis sp. QYM-13
Source: Mar Drugs. 2023 Dec 7;21(12):631. doi: 10.3390/md21120631 (PMC10745014; doi:10.3390/md21120631)
Supplement: Supplementary file 1 [file marinedrugs-21-00631-s001.zip › marinedrugs-2757891-supplementary.pdf]

Cytosporones with anti-inflammatory activities from the mangrove endophytic fungus Phomopsis sp. QYM-13

Guisheng Wang[1,2†], Yilin Yuan[3†], Zhaokun Li[1], Zhigang She[2*] and Yan Chen[1*]

[1]School of Pharmacy, Anhui Medical University, Hefei, 230032, China

[2]School of Chemistry, Sun Yat-sen University, Guangzhou, 510275, China

[3]National R&D Center for Edible Fungus Processing Technology, Henan University, Kaifeng 475004, China

[†]These authors have contributed equally to this work

*Corresponding author

Email address: cychemistry@163.com (Y. Chen); cesshzhg@mail.sysu.edu.cn (Z. She).

Supporting Information Contents:

Figure. S1 ^{1}H NMR spectrum of compound **1** (500 MHz, MeOD-d_4).

Figure. S2 ^{13}C NMR spectrum of compound **1** (125 MHz, MeOD-d_4).

Figure. S3 HSQC spectrum of compound **1**.

Figure. S4 ^{1}H-^{1}H COSY spectrum of compound **1**.

Figure. S5 HMBC spectrum of compound **1**.

Figure. S6 HRESIMS spectrum of compound **1**.

Figure. S7 ^{1}H NMR spectrum of compound **2** (500 MHz, MeOD-d_4).

Figure. S8 ^{13}C NMR spectrum of compound **2** (125 MHz, MeOD-d_4).

Figure. S9 HSQC spectrum of compound **2**.

Figure. S10 ^{1}H-^{1}H COSY spectrum of compound **2**.

Figure. S11 HMBC spectrum of compound **2**.

Figure. S12 HRESIMS spectrum of compound **2**.

Figure. S13 ^{1}H NMR spectrum of compound **3** (500 MHz, MeOD-d_4).

Figure. S14 ^{13}C NMR spectrum of compound **3** (125 MHz, MeOD-d_4).

Figure. S15 HSQC spectrum of compound **3**.

Figure. S16 ^{1}H-^{1}H COSY spectrum of compound **3**.

Figure. S17 HMBC spectrum of compound **3**.

Figure. S18 HRESIMS spectrum of compound **3**.

Figure. S19 ^{1}H NMR spectrum of compound **4** (500 MHz, MeOD-d_4).

Figure. S20 ^{13}C NMR spectrum of compound **4** (125 MHz, MeOD-d_4).

Figure. S21 HSQC spectrum of compound **4**.

Figure. S22 ^{1}H-^{1}H COSY spectrum of compound **4**.

Figure. S23 HMBC spectrum of compound **4**.

Figure. S24 HRESIMS spectrum of compound **4**.

Figure. S25 ^{1}H NMR spectrum of compound **5** (500 MHz, MeOD-d_4).

Figure. S26 ^{13}C NMR spectrum of compound **5** (125 MHz, MeOD-d_4).

Figure. S27 HSQC spectrum of compound **5**.

Figure. S28 ^{1}H-^{1}H COSY spectrum of compound **5**.

Figure. S29 HMBC spectrum of compound **5**.

Figure. S30 HRESIMS spectrum of compound **5**.

Figure. S31 ^{1}H NMR spectrum of compound **13** (500 MHz, DMSO-d_6).

Figure. S32 ^{13}C NMR spectrum of compound **13** (125 MHz, DMSO-d_6).

Figure. S33 HSQC spectrum of compound **13**.

Figure. S34 ^{1}H-^{1}H COSY spectrum of compound **13**.

Figure. S35 HMBC spectrum of compound **13**.

Figure. S36 HRESIMS spectrum of compound **13**.

Figure. S37 ^{1}H NMR spectrum of compound **14** (500 MHz, DMSO-d_6).

Figure. S38 ^{13}C NMR spectrum of compound **14** (125 MHz, DMSO-d_6).

Figure. S39 HSQC spectrum of compound **14**.

Figure. S40 ^{1}H-^{1}H COSY spectrum of compound **14**.

Figure. S41 HMBC spectrum of compound **14**.

Figure. S42 NOESY spectrum of compound **14**.

Figure. S43 HRESIMS spectrum of compound **14**.

Figure. S44 ^{1}H NMR spectrum of compound **15** (500 MHz, DMSO-d_6).

Figure. S45 ^{13}C NMR spectrum of compound **15** (125 MHz, DMSO-d_6).

Figure. S46 HSQC spectrum of compound **15**.

Figure. S47 ^{1}H-^{1}H COSY spectrum of compound **15**.

Figure. S48 HMBC spectrum of compound **15**.

Figure. S49 NOESY spectrum of compound **15**.

Figure. S50 HRESIMS spectrum of compound **15**.

Table S1. The DP4+ evaluation of compound **14**.

Table S2. The binding energy of compounds **1-13** with numerous inflammatory targets (kcal/mol).

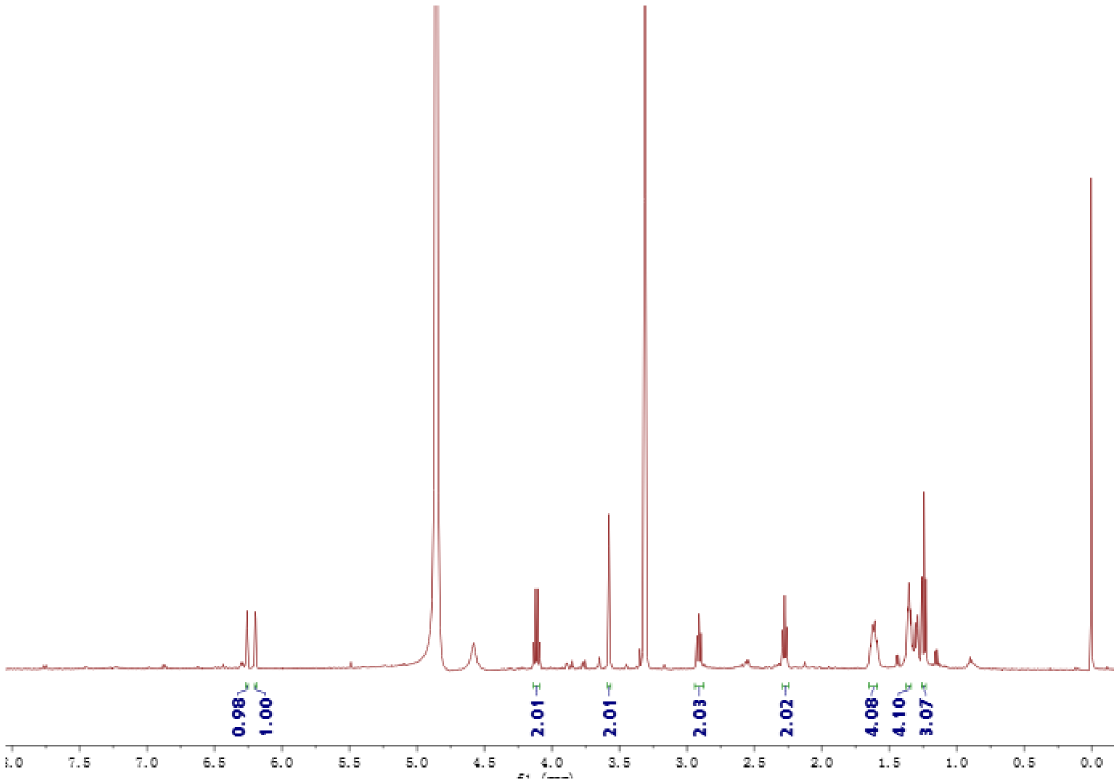

Figure. S1 ^{1}H NMR spectrum of compound **1** (500 MHz, MeOD-d_4).

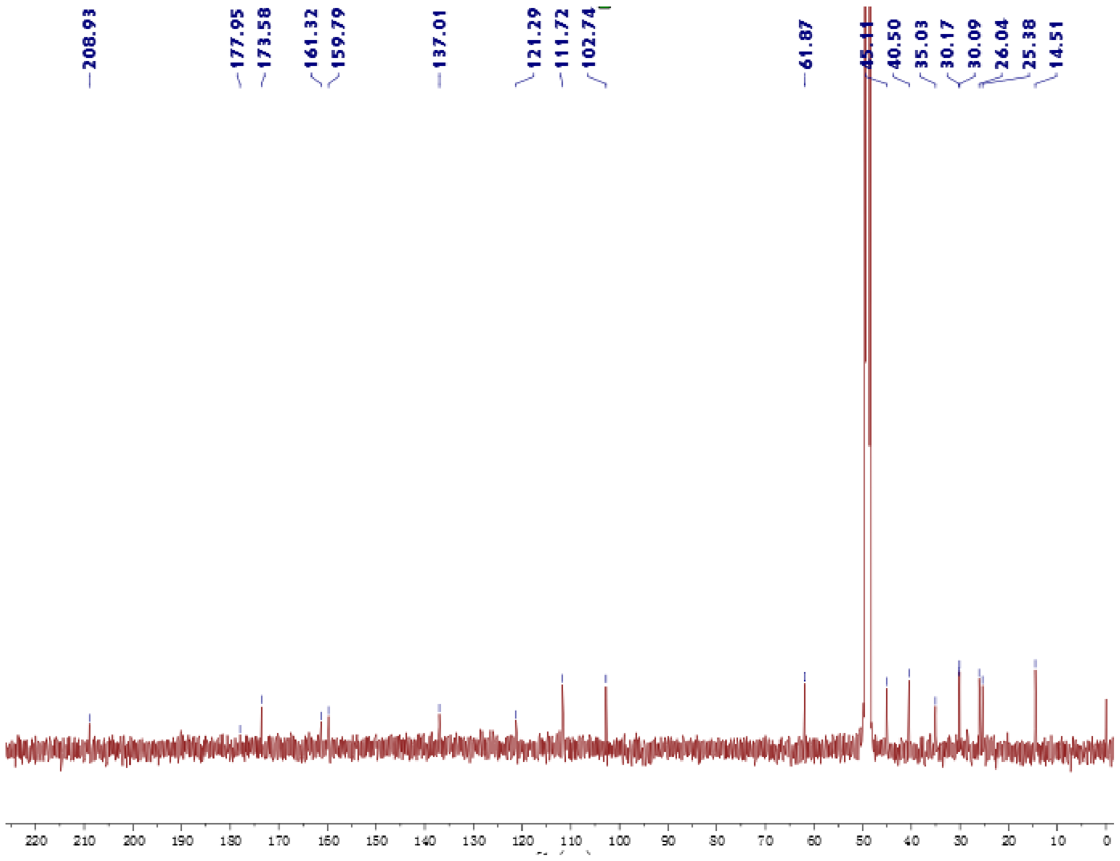

Figure. S2 ^{13}C NMR spectrum of compound **1** (125 MHz, MeOD-d_4).

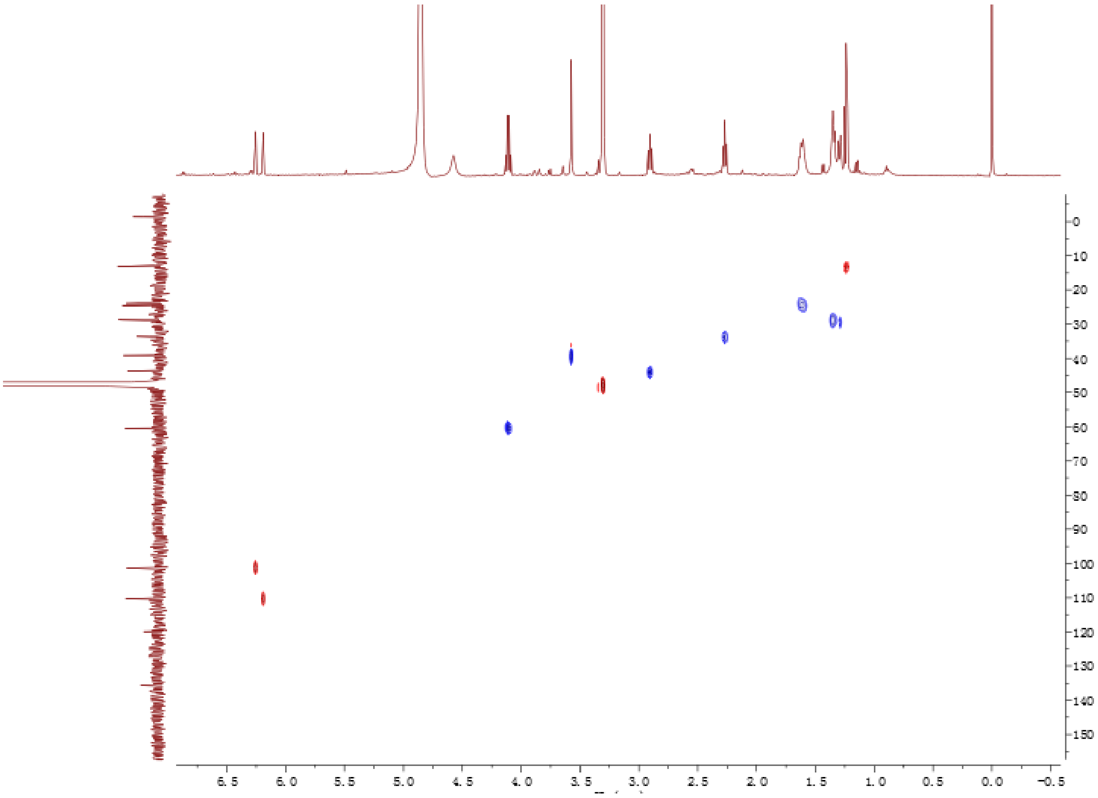

Figure. S3 HSQC spectrum of compound **1**.

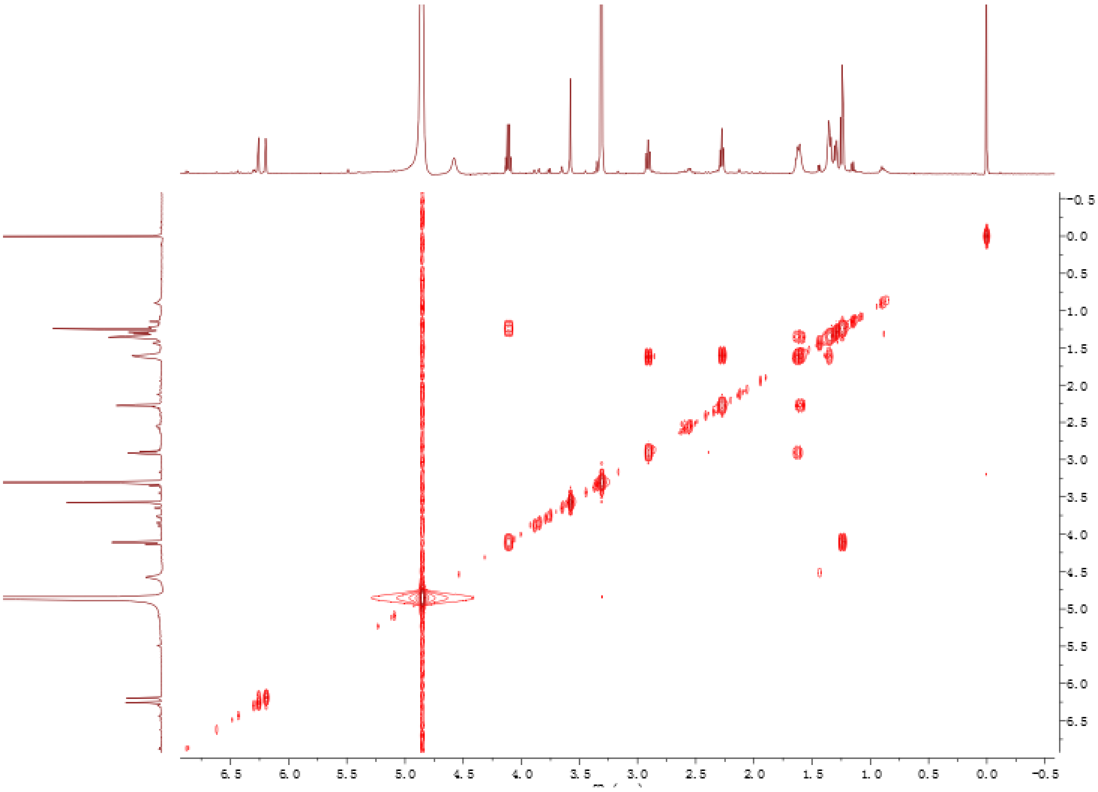

Figure. S4 ^{1}H-^{1}H COSY spectrum of compound **1**.

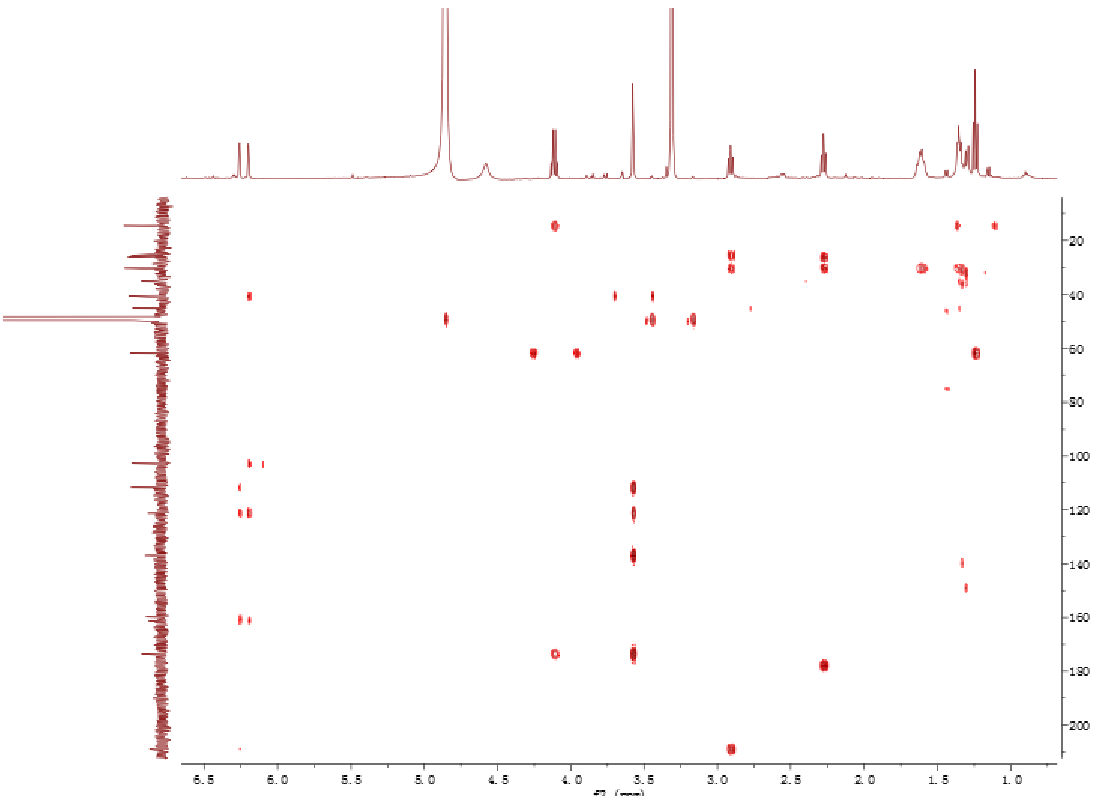

Figure. S5 HMBC spectrum of compound **1**.

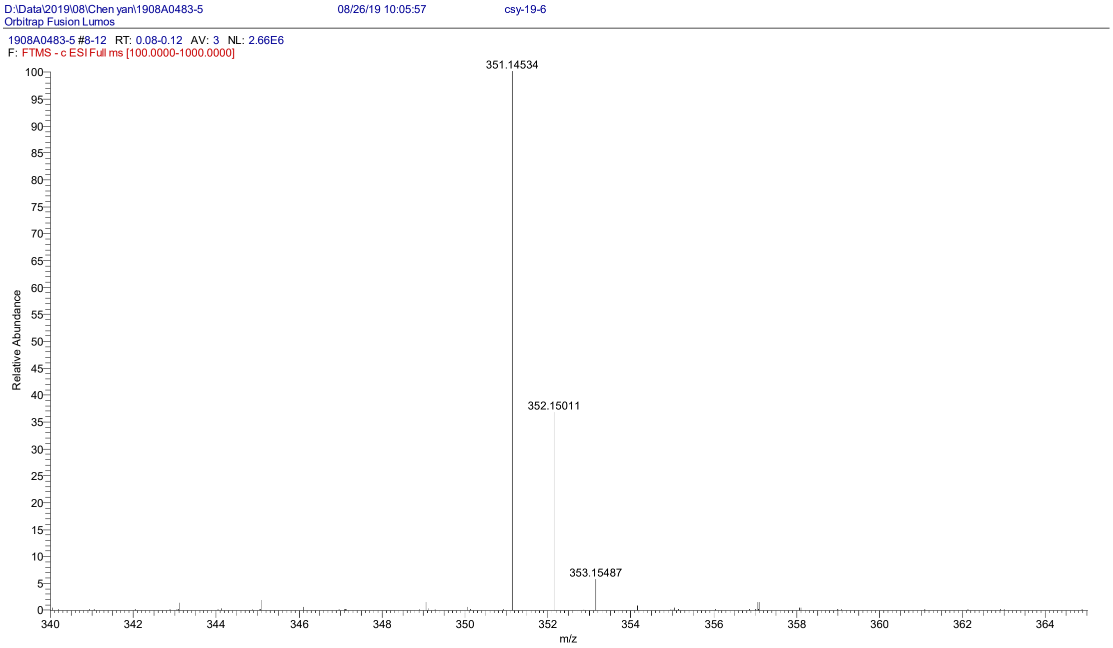

Figure. S6 HRESIMS spectrum of compound **1**.

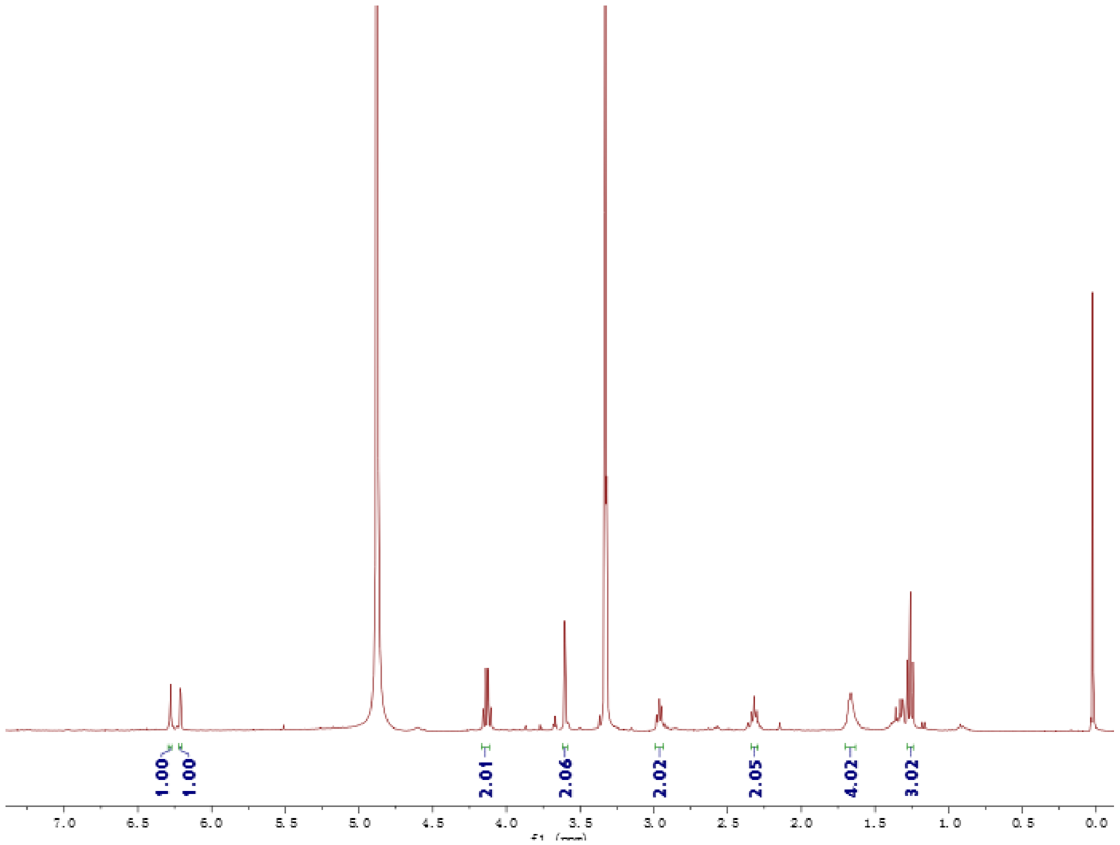

Figure. S7 ^{1}H NMR spectrum of compound **2** (500 MHz, MeOD-d_4).

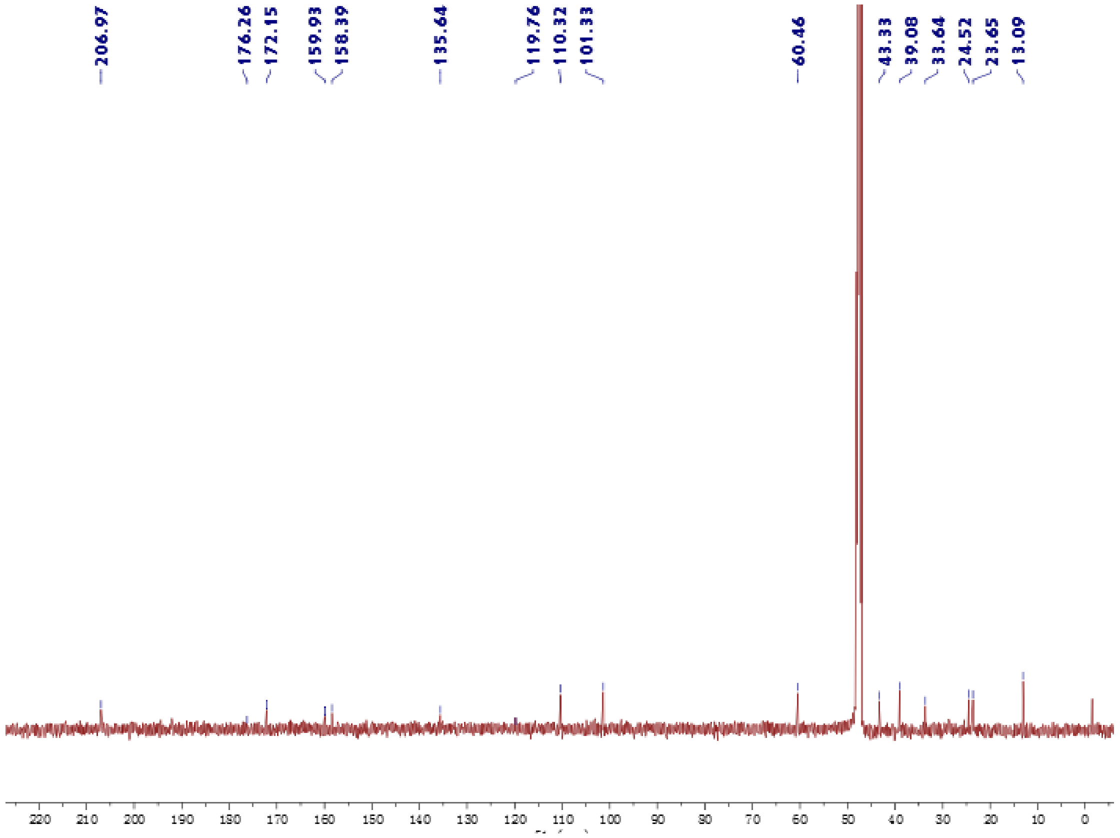

Figure. S7 ^{1}H NMR spectrum of compound **2** (500 MHz, MeOD-d_4).

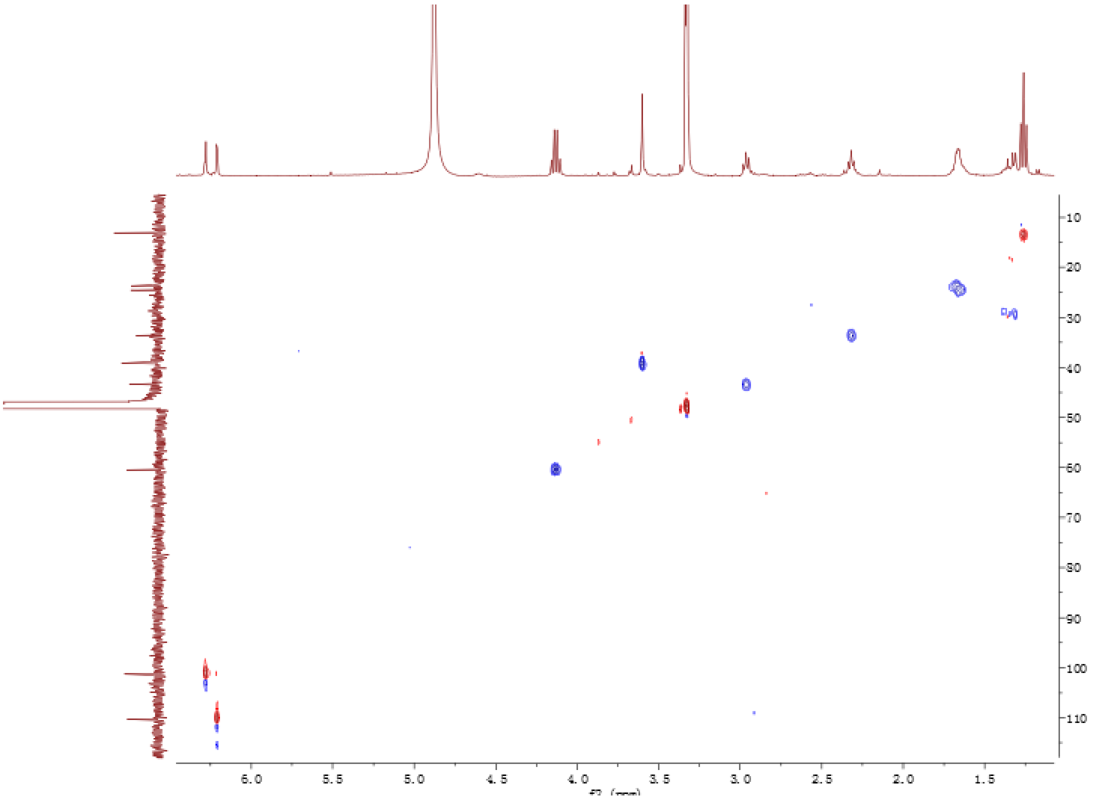

Figure. S9 HSQC spectrum of compound **2**.

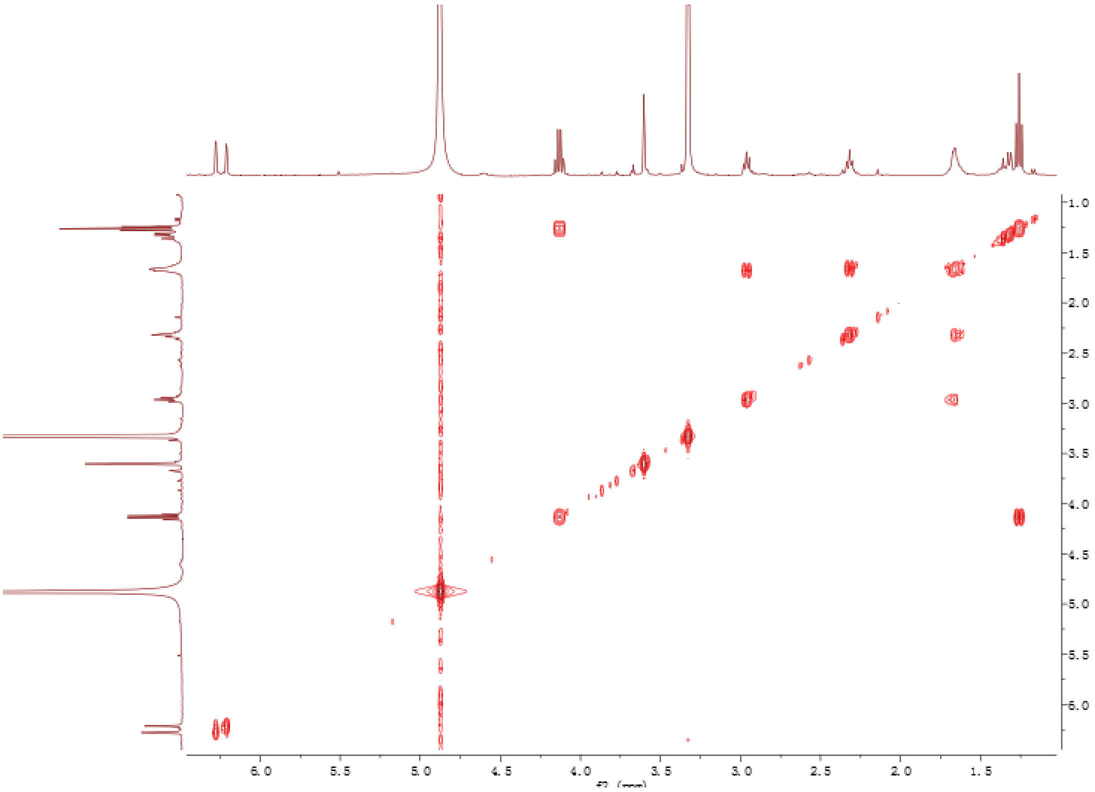

Figure. S10 ^{1}H-^{1}H COSY spectrum of compound **2**.

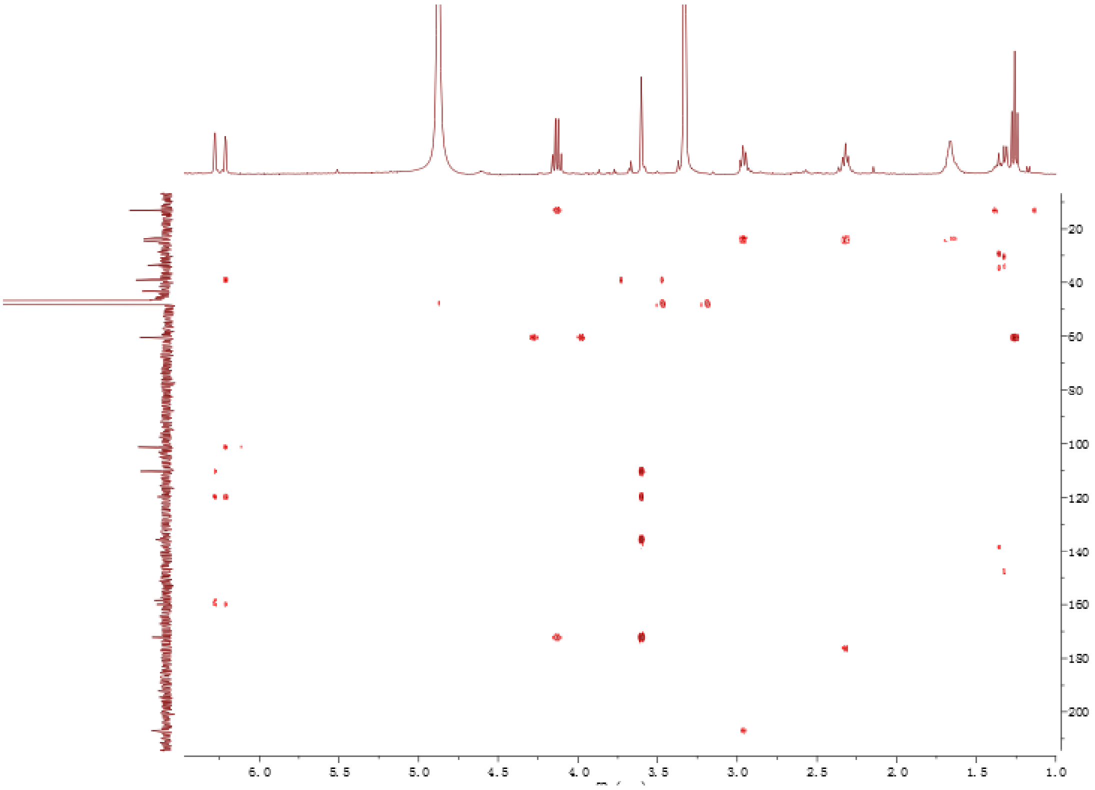

Figure. S11 HMBC spectrum of compound **2**.

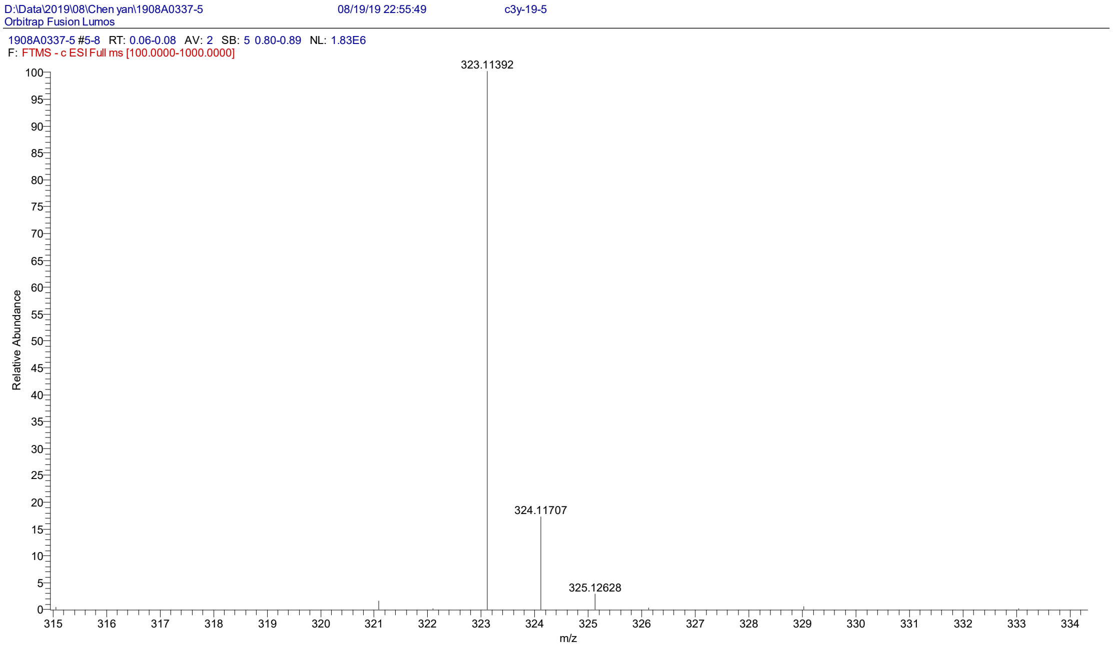

Figure. S12 HRESIMS spectrum of compound **2**.

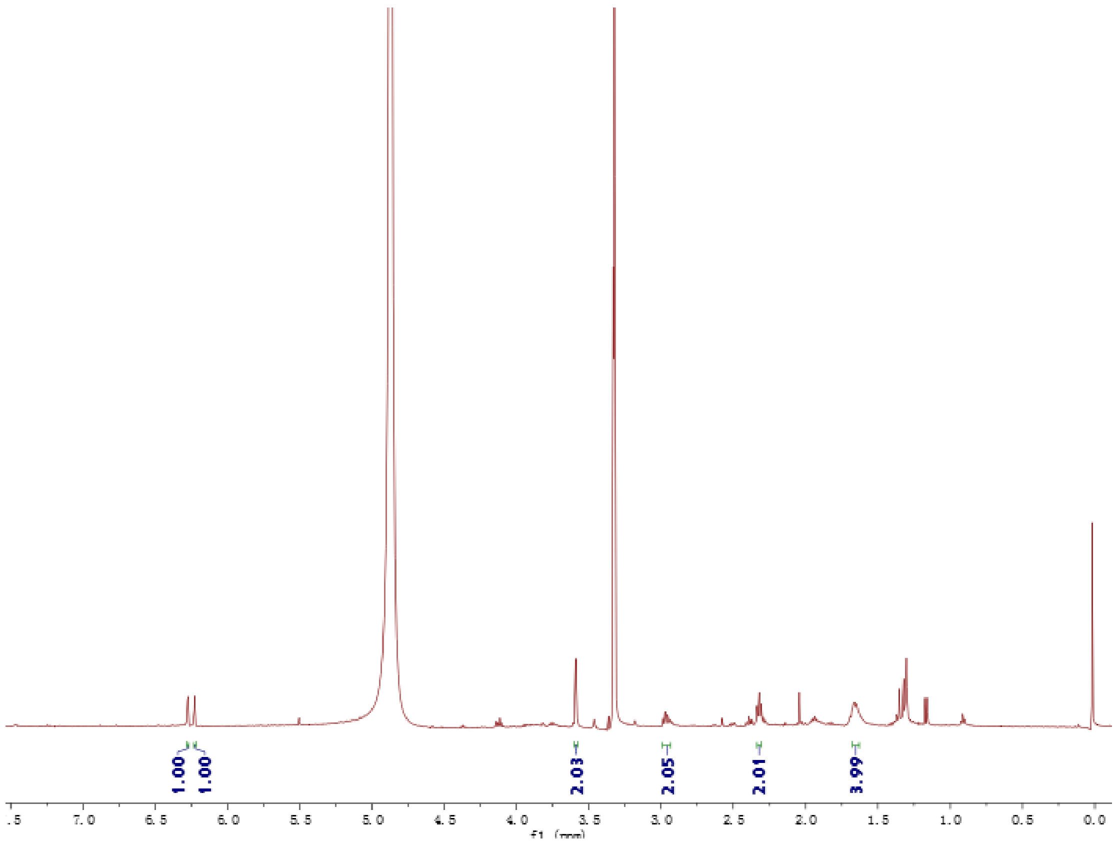

Figure. S13 ^{1}H NMR spectrum of compound **3** (500 MHz, MeOD-d_4).

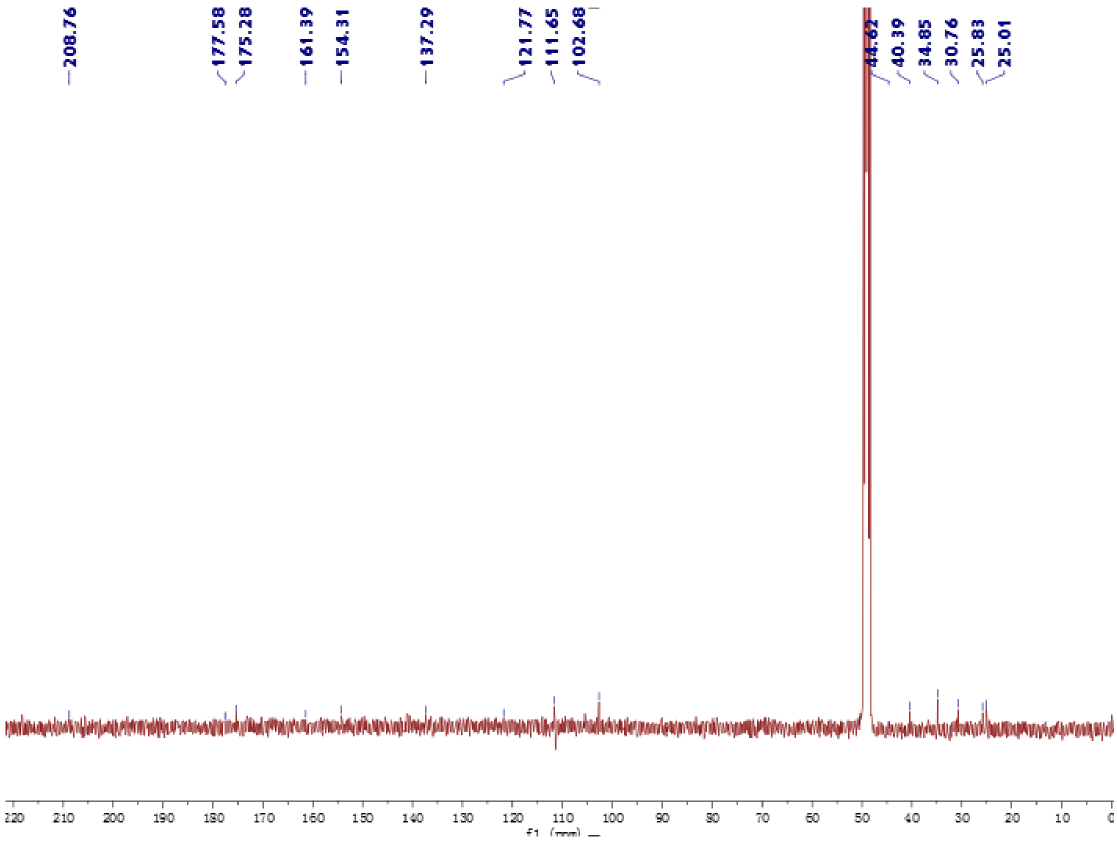

Figure. S14 ^{13}C NMR spectrum of compound **3** (125 MHz, MeOD-d_4).

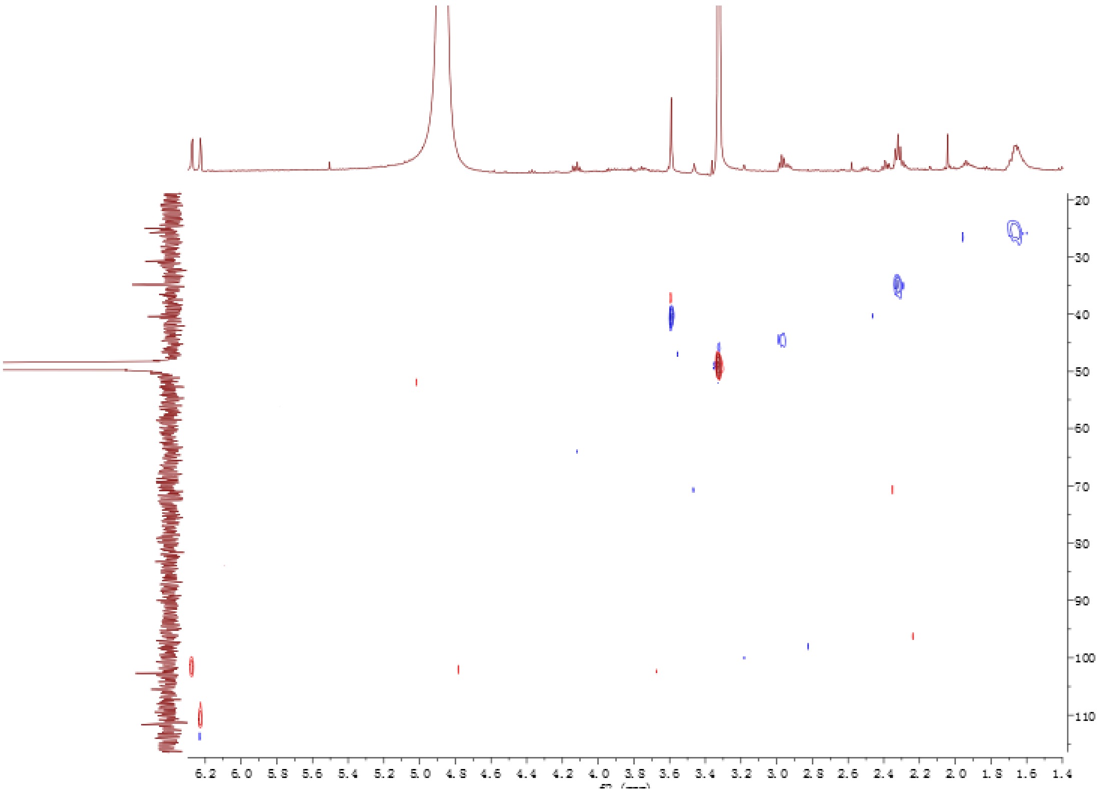

Figure. S15 HSQC spectrum of compound **3**.

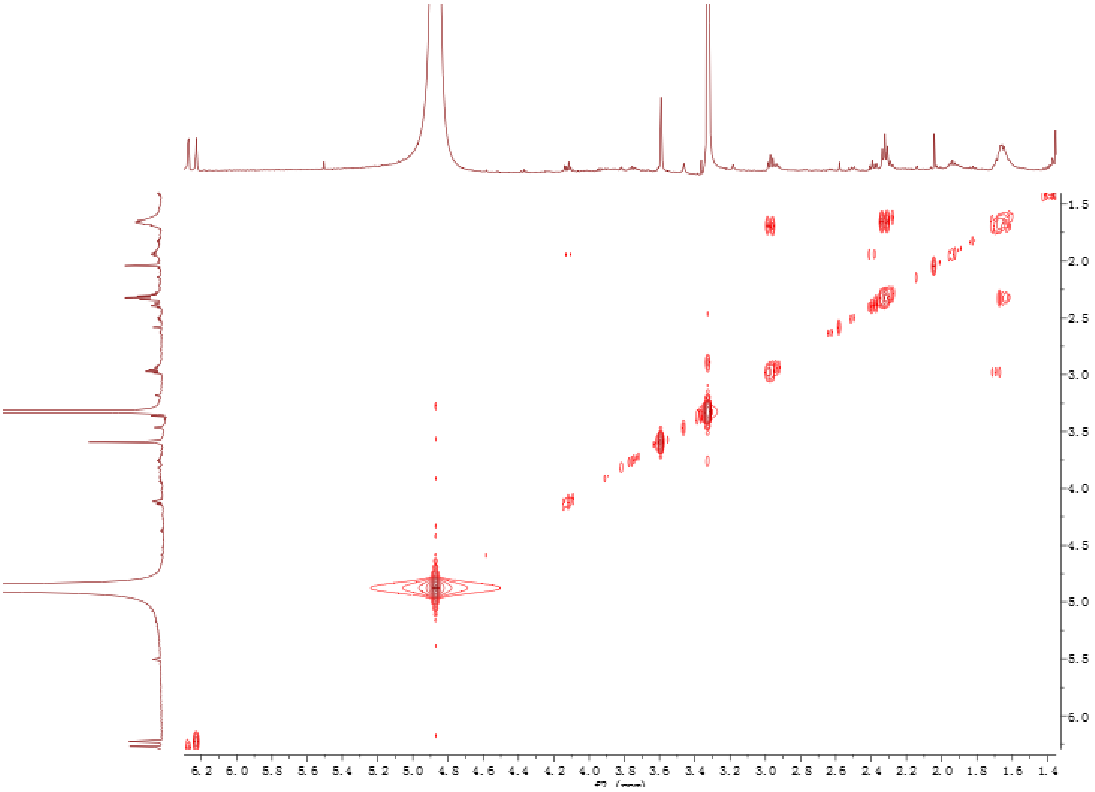

Figure. S16 ^{1}H-^{1}H COSY spectrum of compound **3**.

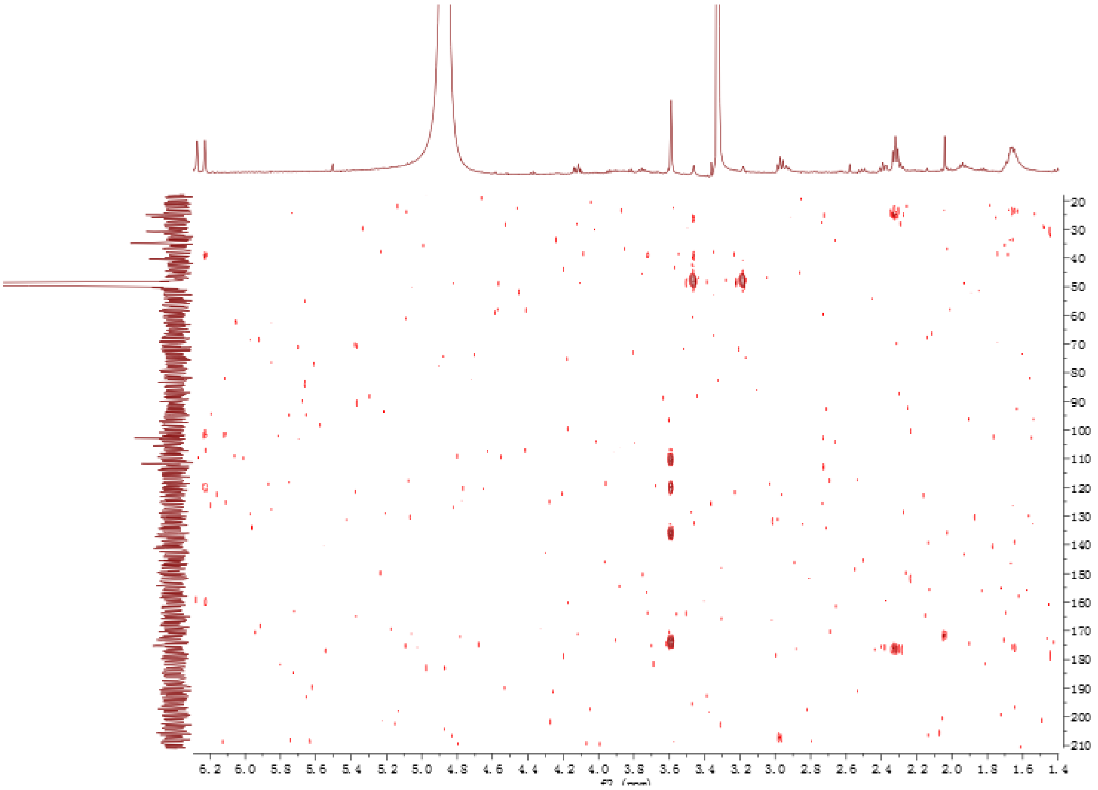

Figure. S17 HMBC spectrum of compound **3**.

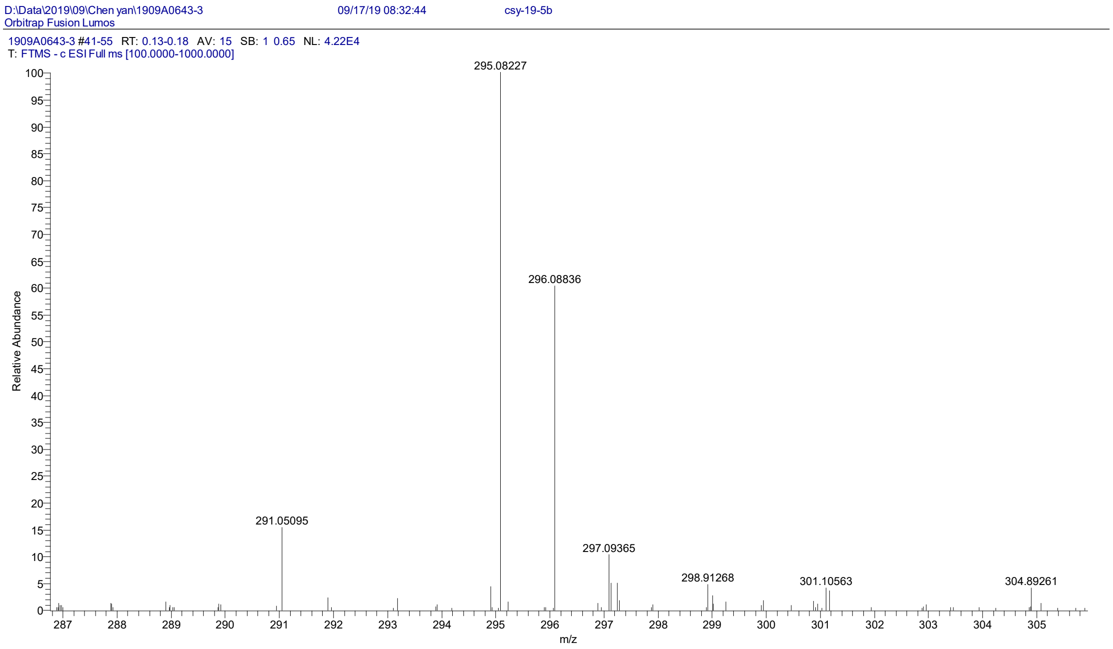

Figure. S18 HRESIMS spectrum of compound **3**.

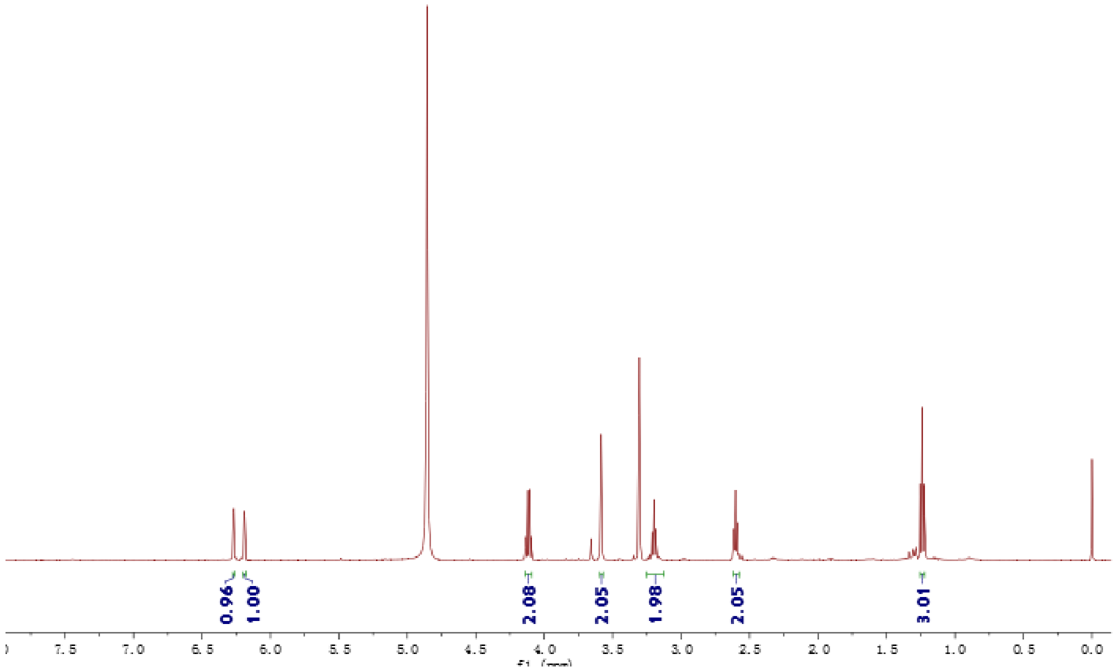

Figure. S19 ^{1}H NMR spectrum of compound **4** (500 MHz, MeOD-d_4).

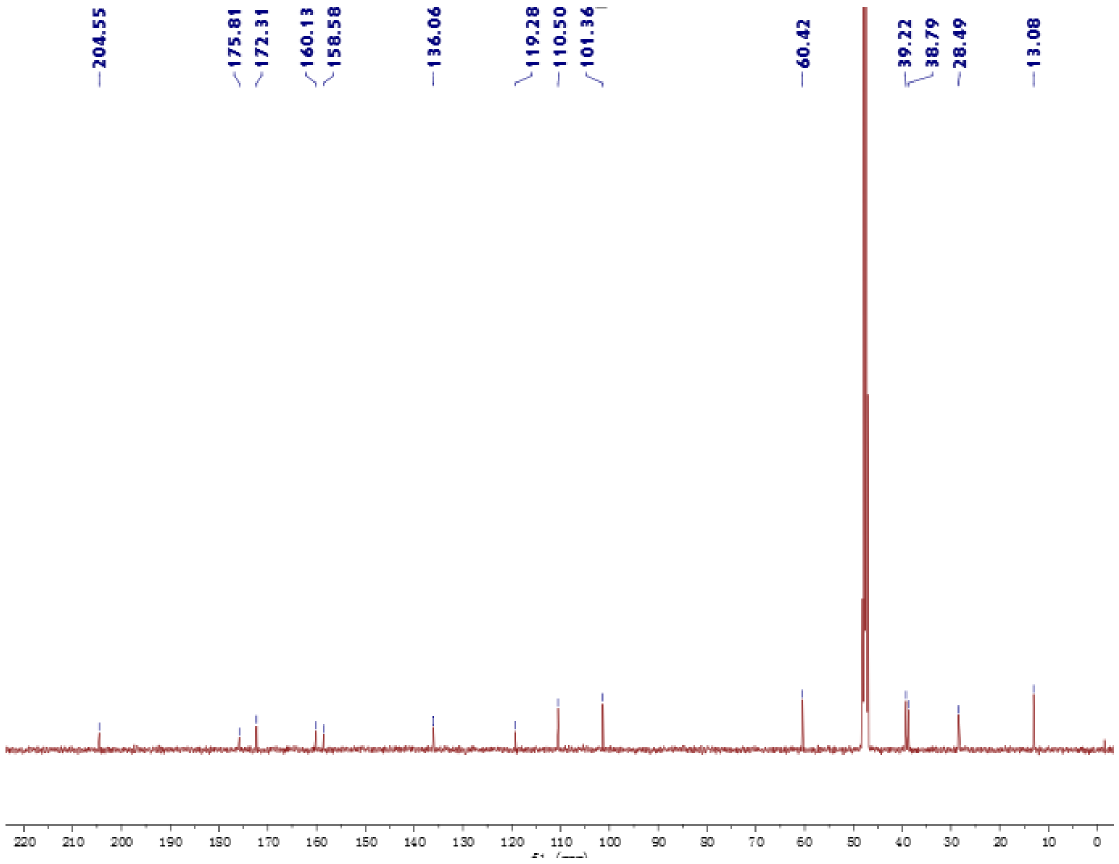

Figure. S20 ^{13}C NMR spectrum of compound **4** (125 MHz, MeOD-d_4).

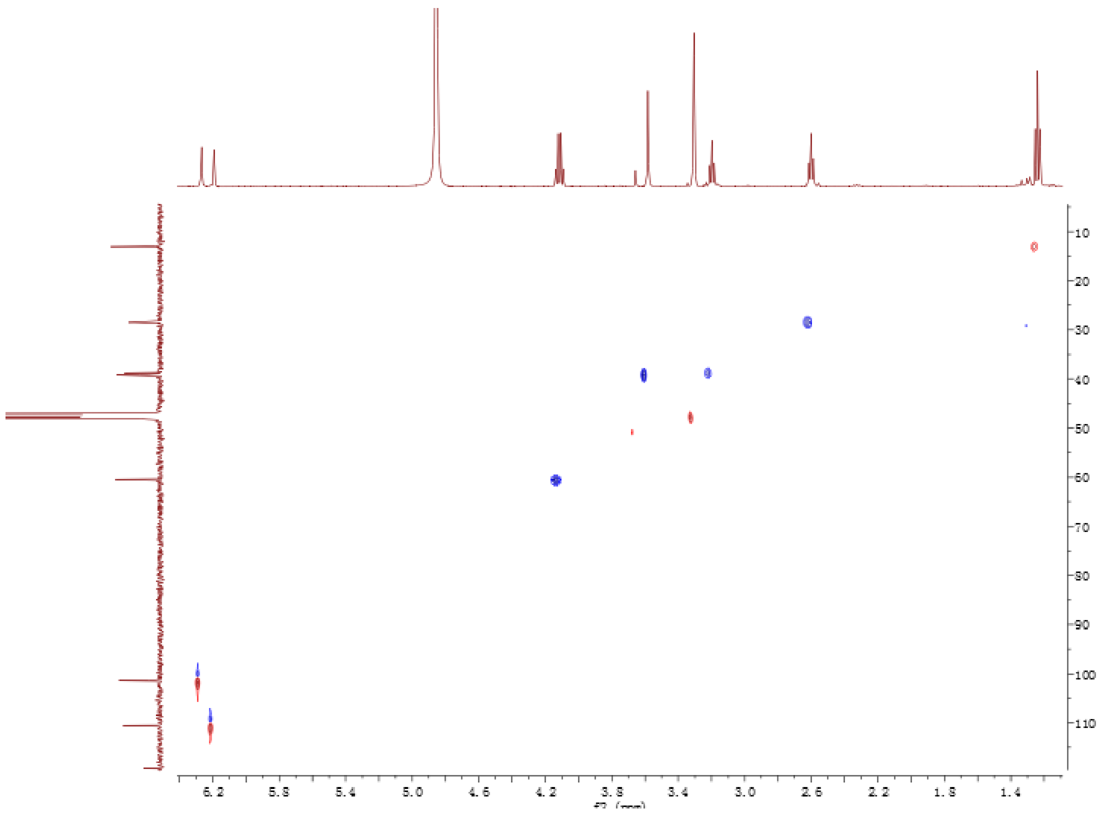

Figure. S21 HSQC spectrum of compound **4**.

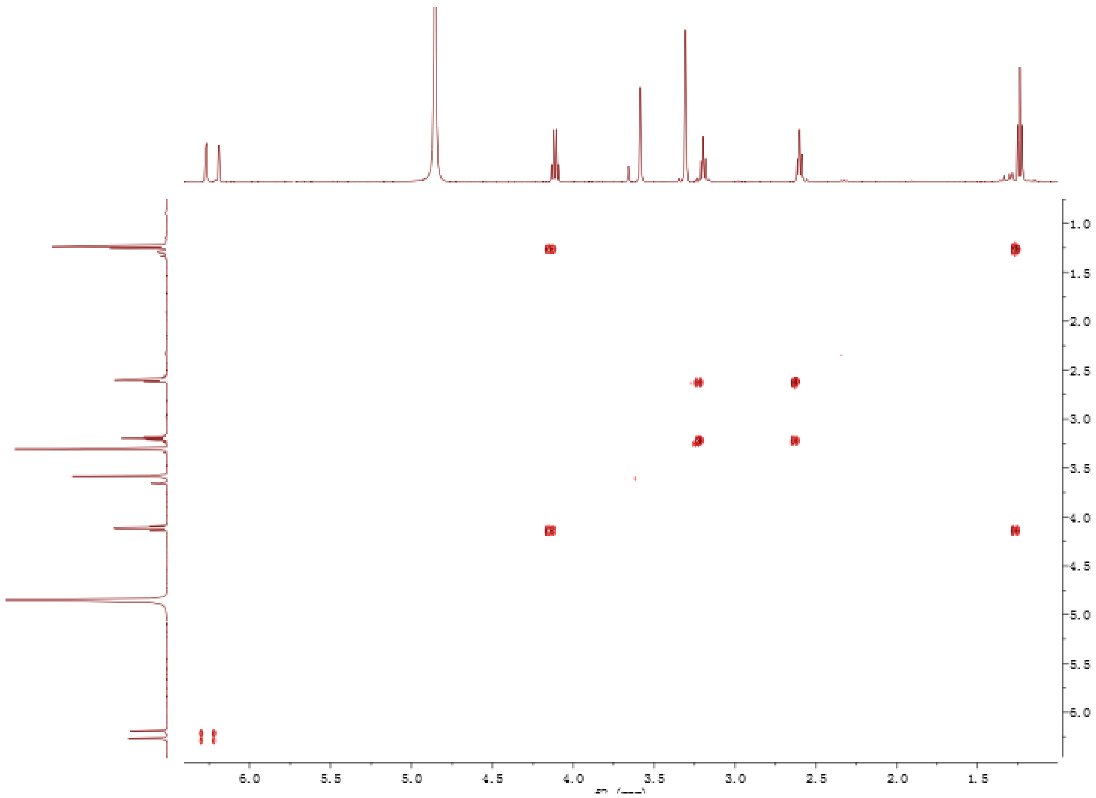

Figure. S22 ^{1}H-^{1}H COSY spectrum of compound **4**.

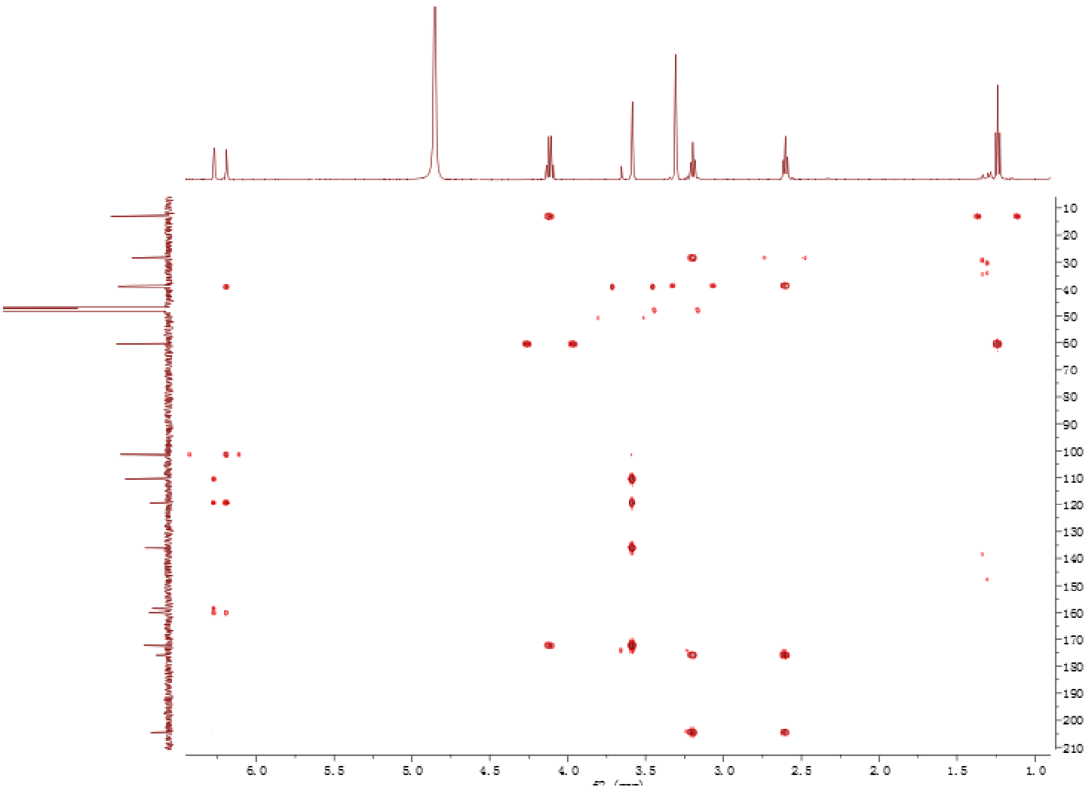

Figure. S23 HMBC spectrum of compound **4**.

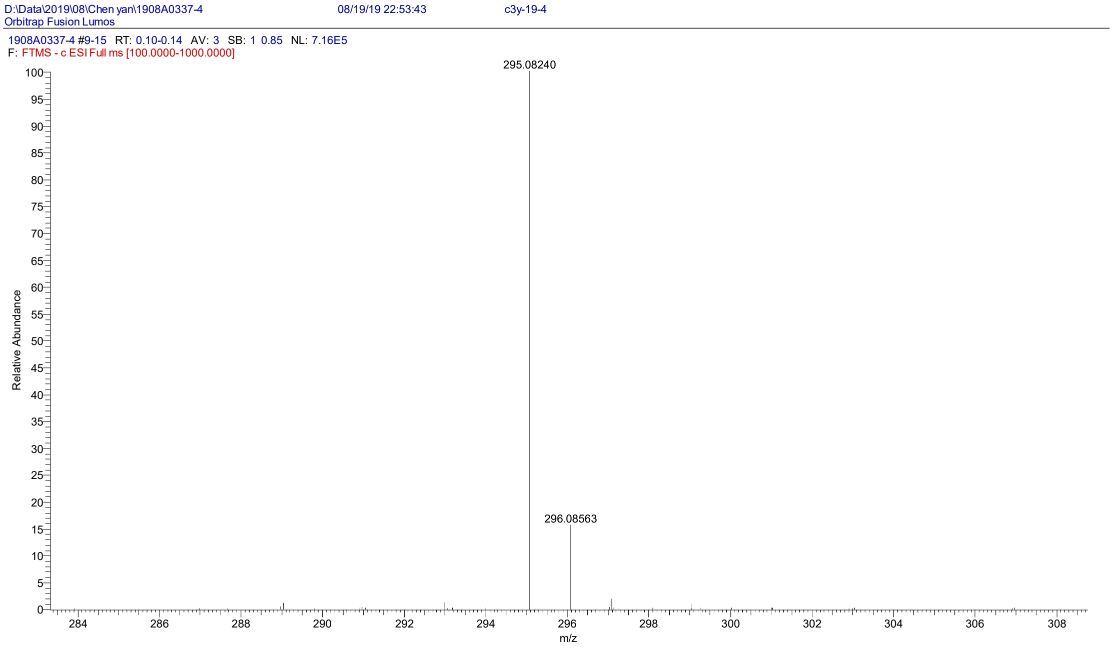

Figure. S24 HRESIMS spectrum of compound **4**.

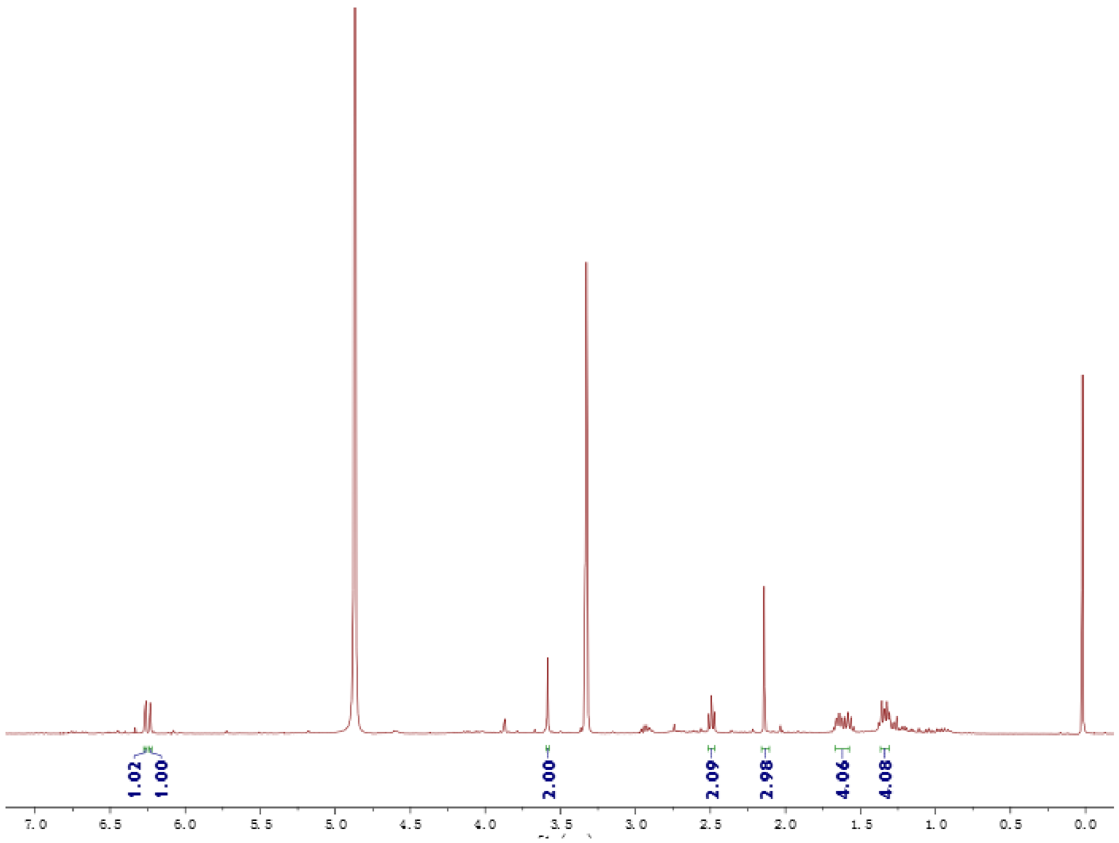

Figure. S25 ^{1}H NMR spectrum of compound **5** (500 MHz, MeOD-d_4).

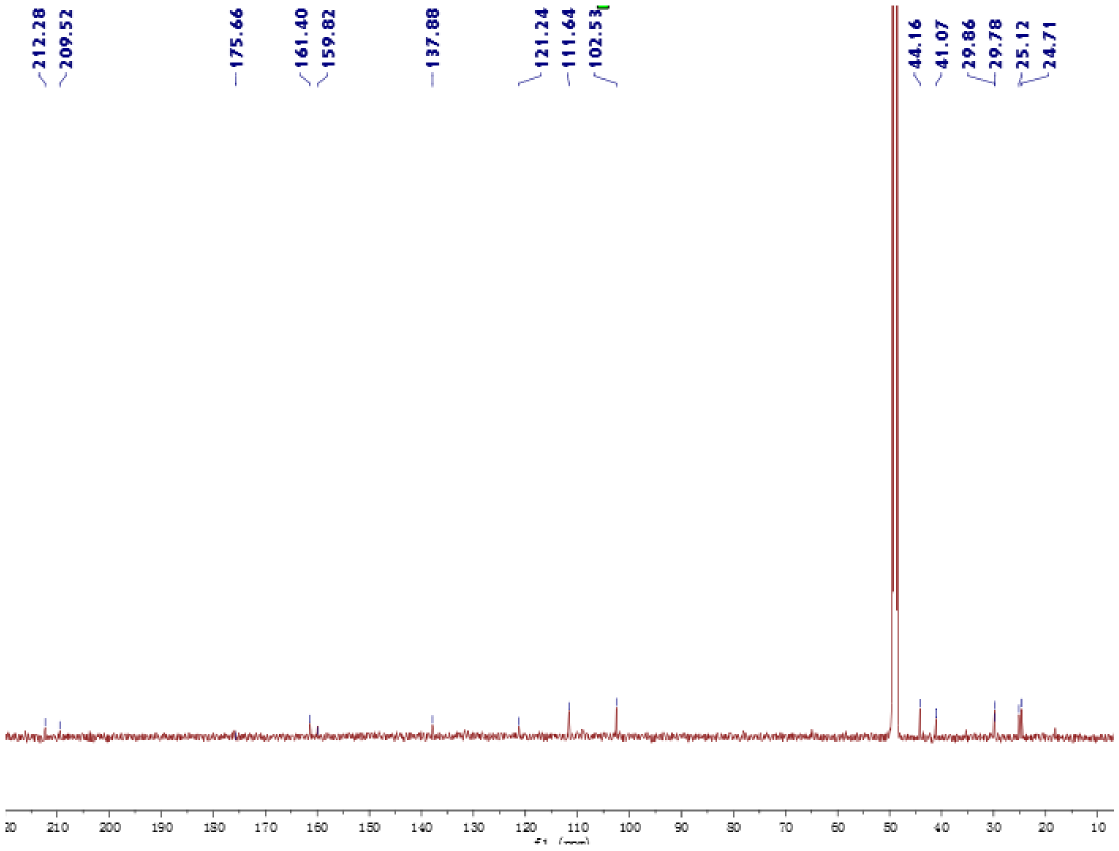

Figure. S26 ^{13}C NMR spectrum of compound **5** (125 MHz, MeOD-d_4).

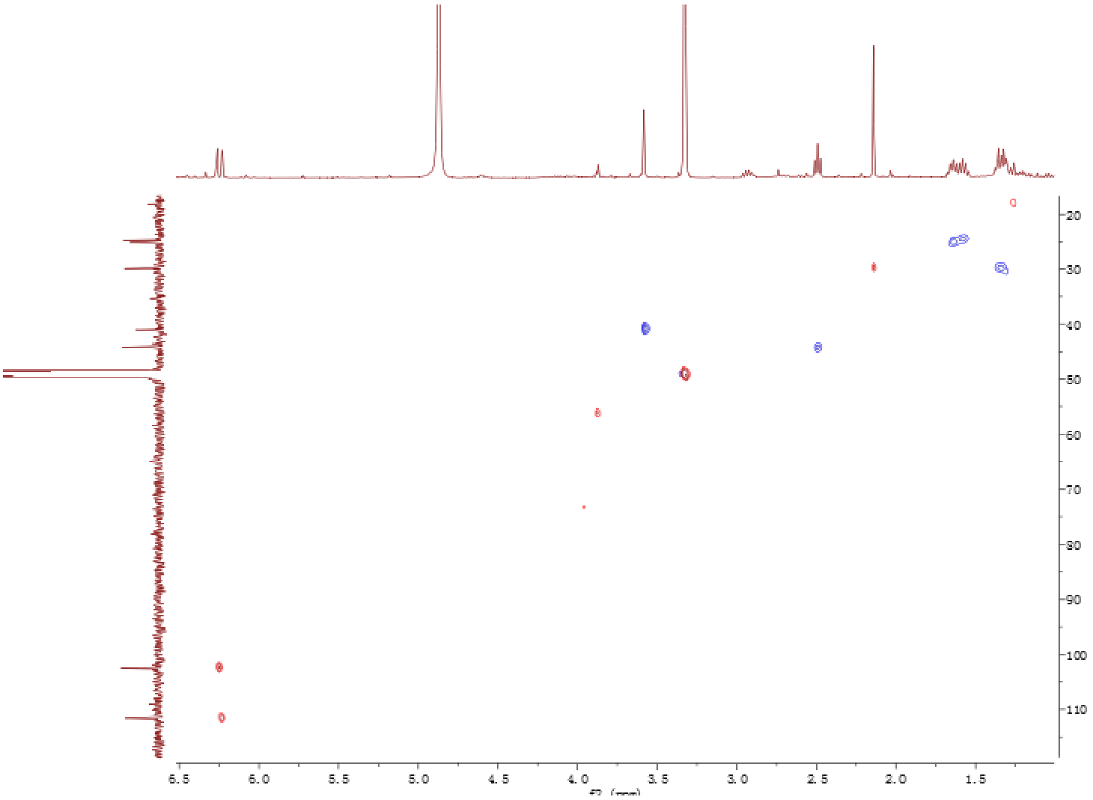

Figure. S27 HSQC spectrum of compound **5**.

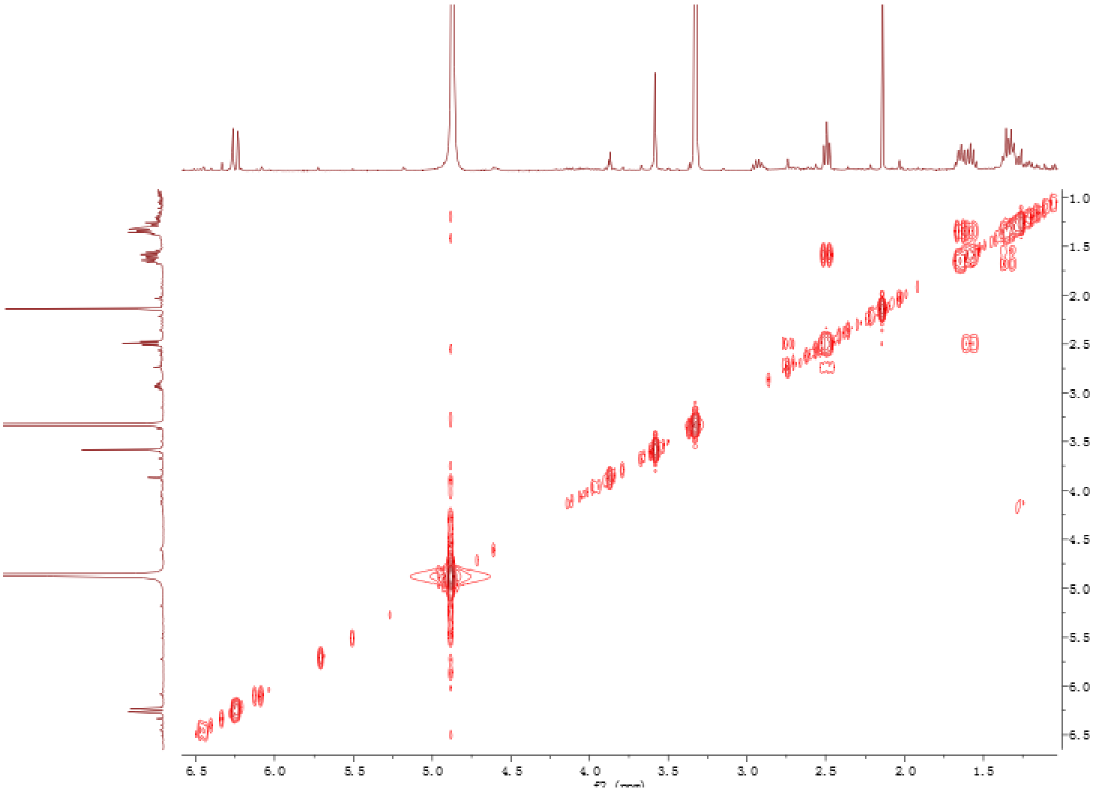

Figure. S28 ^{1}H-^{1}H COSY spectrum of compound **5**.

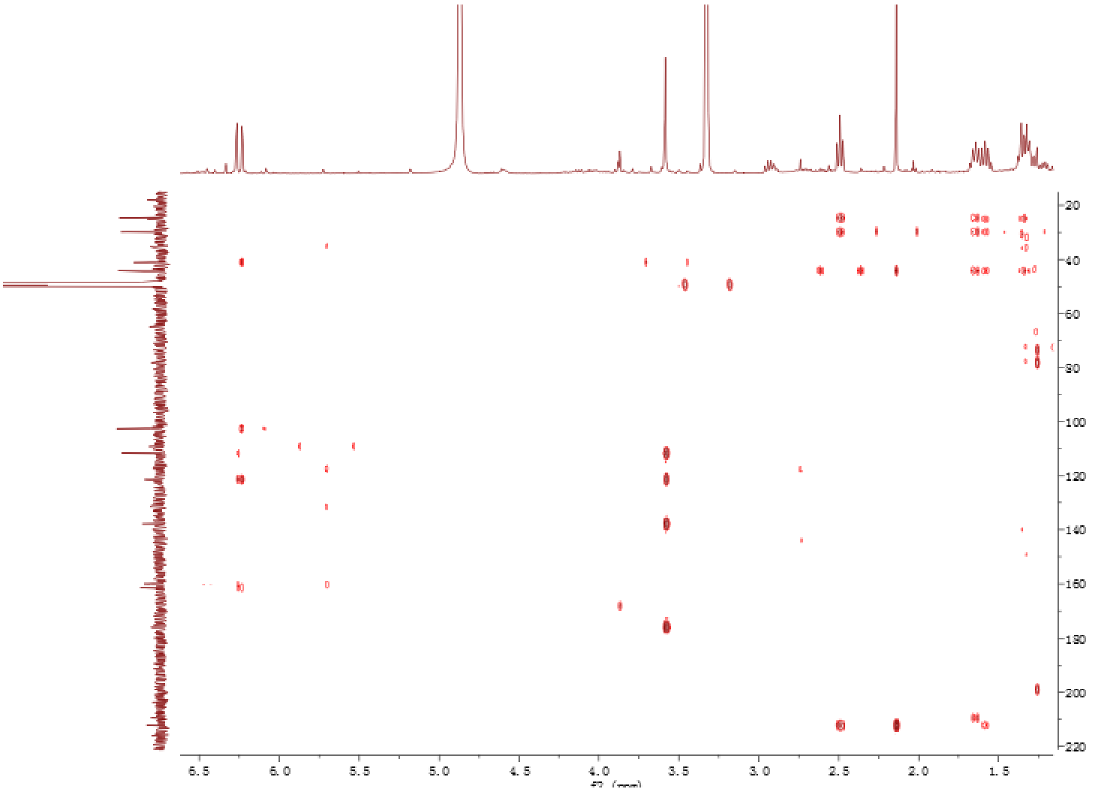

Figure. S29 HMBC spectrum of compound **5**.

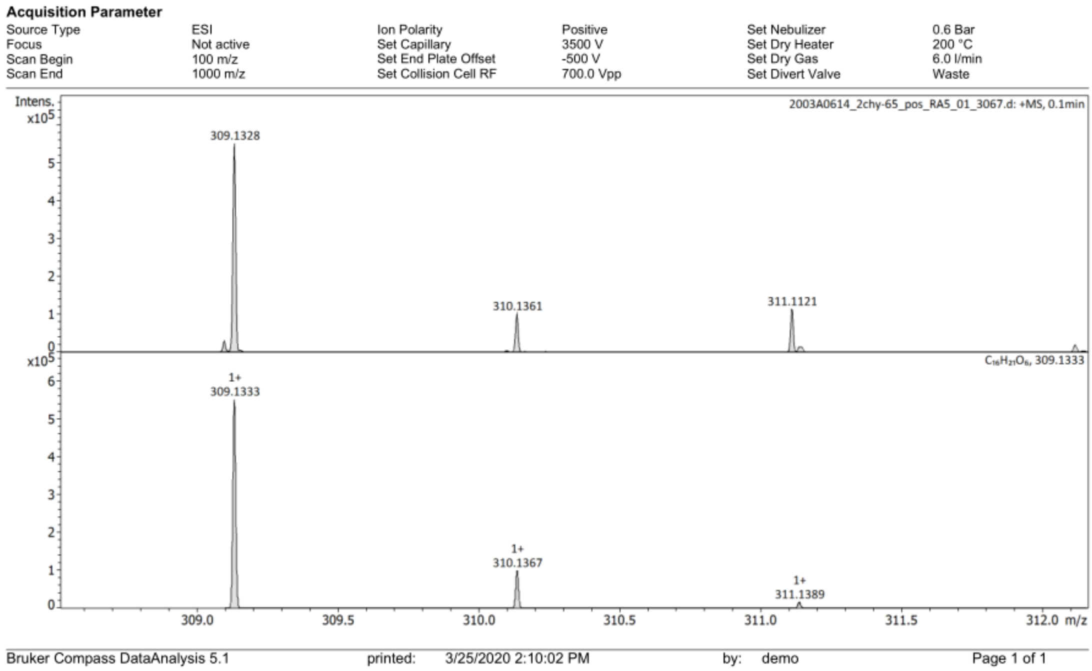

Figure. S30 HRESIMS spectrum of compound **5**.

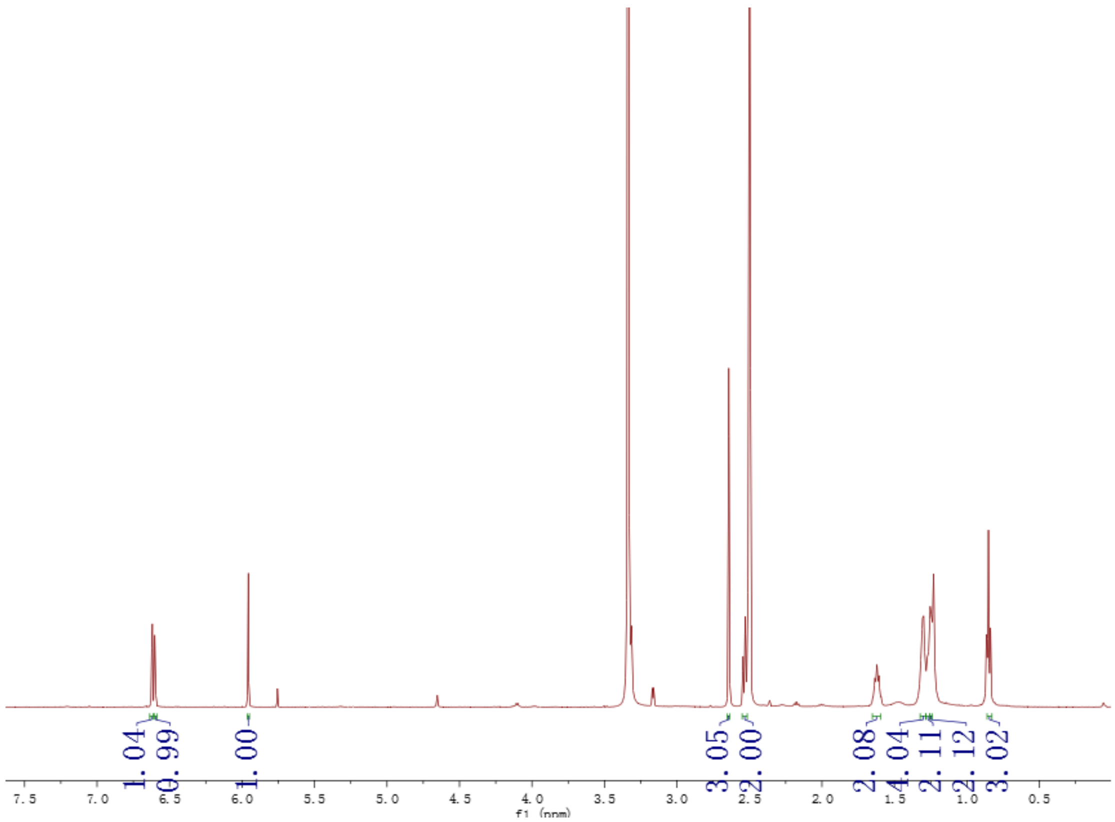

Figure. S31 ^{1}H NMR spectrum of compound **13** (500 MHz, DMSO-d_6).

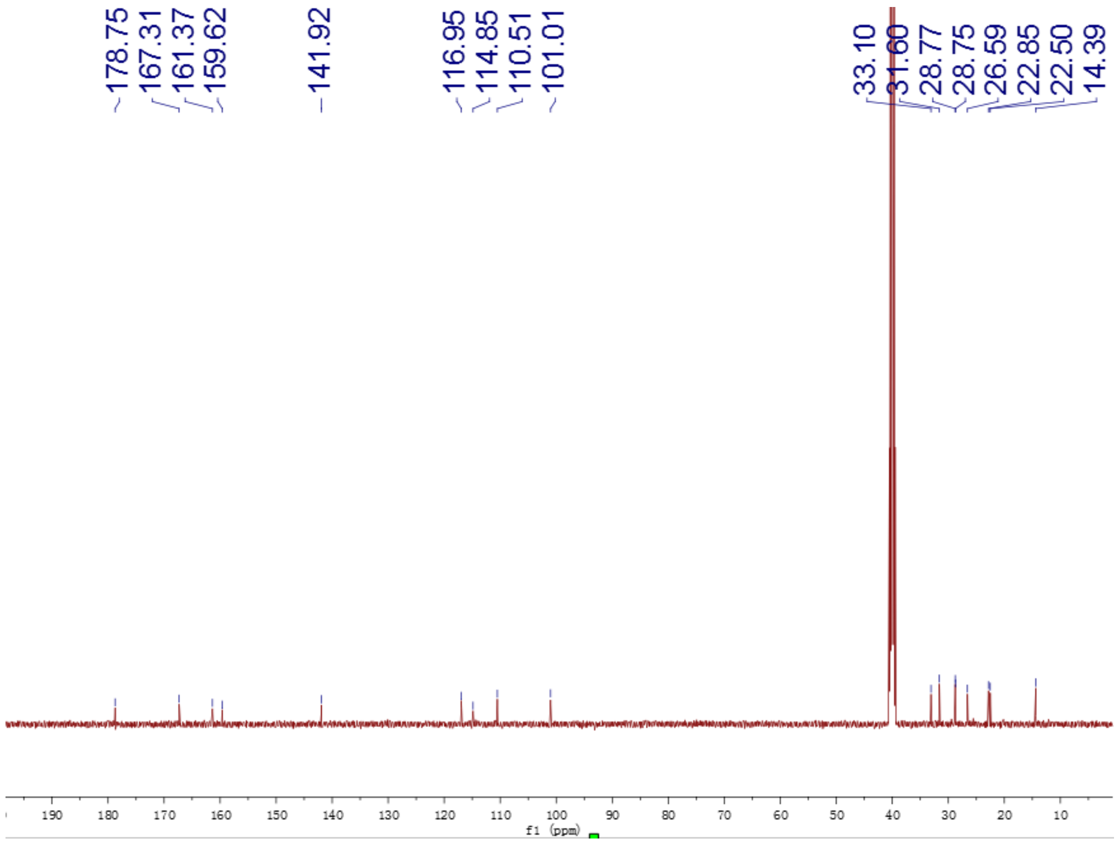

Figure. S32 ^{13}C NMR spectrum of compound **13** (125 MHz, DMSO-d_6).

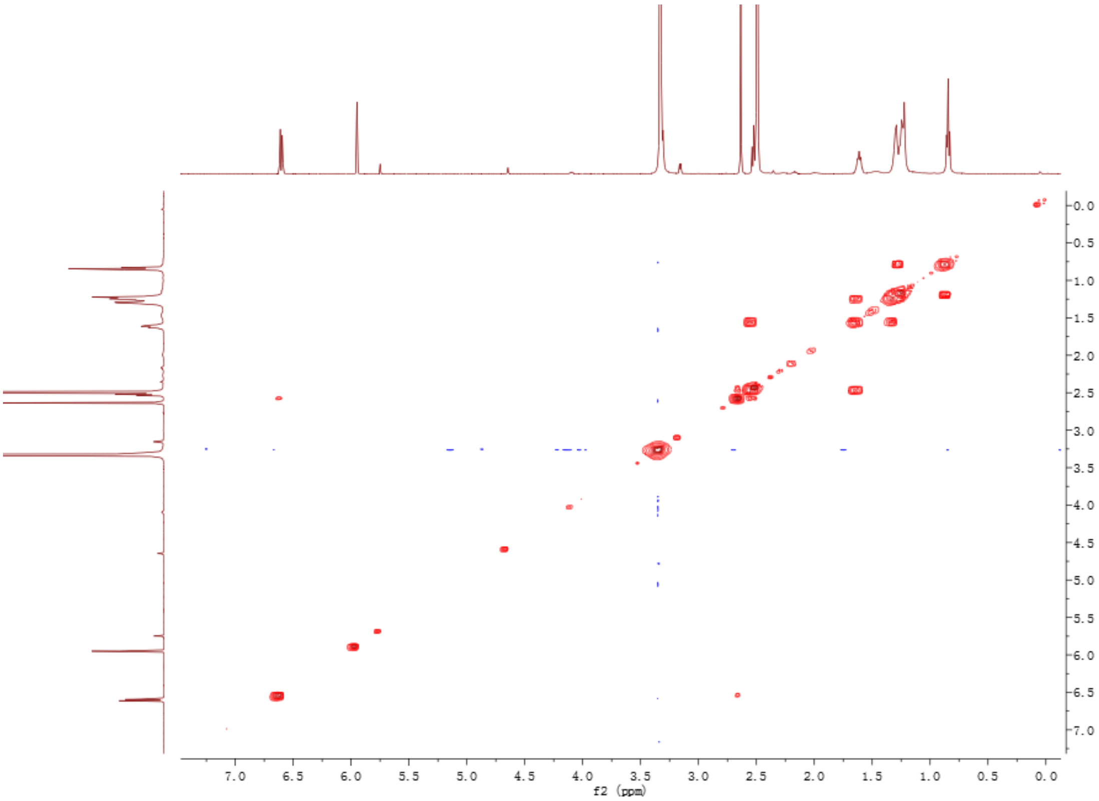

Figure. S33 HSQC spectrum of compound **13**.

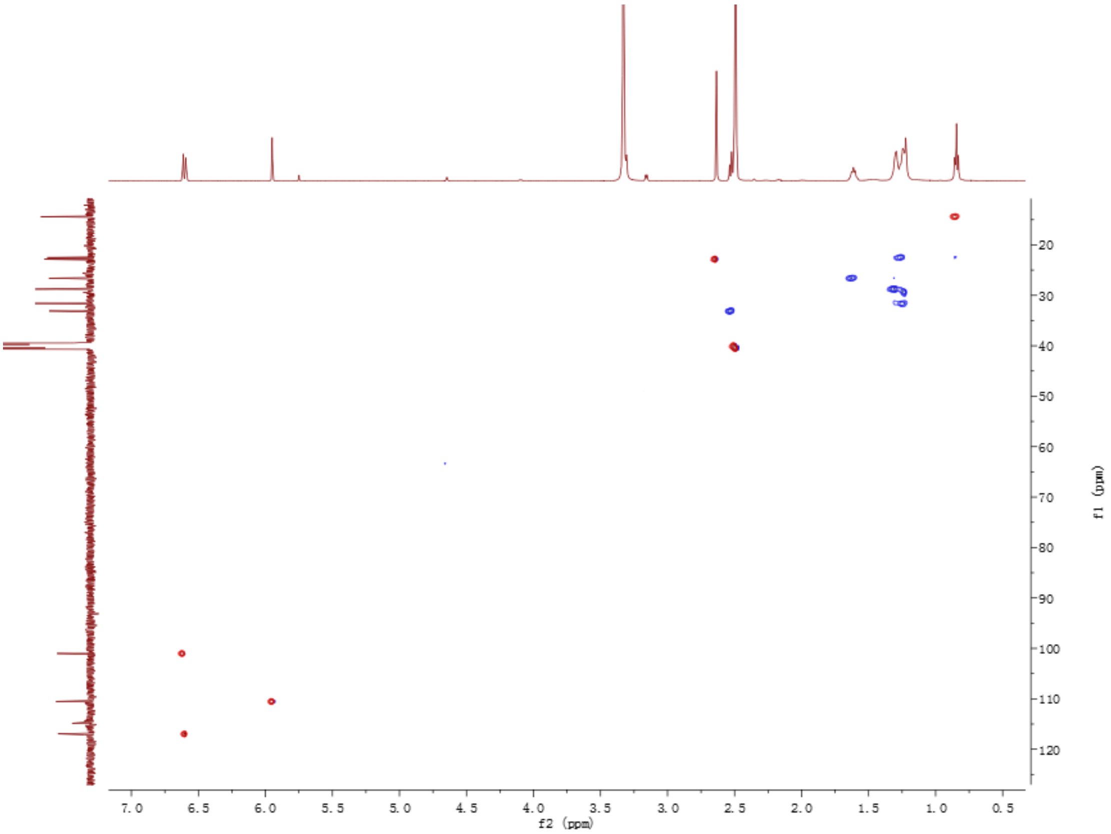

Figure. S34 ^{1}H-^{1}H COSY spectrum of compound **13**.

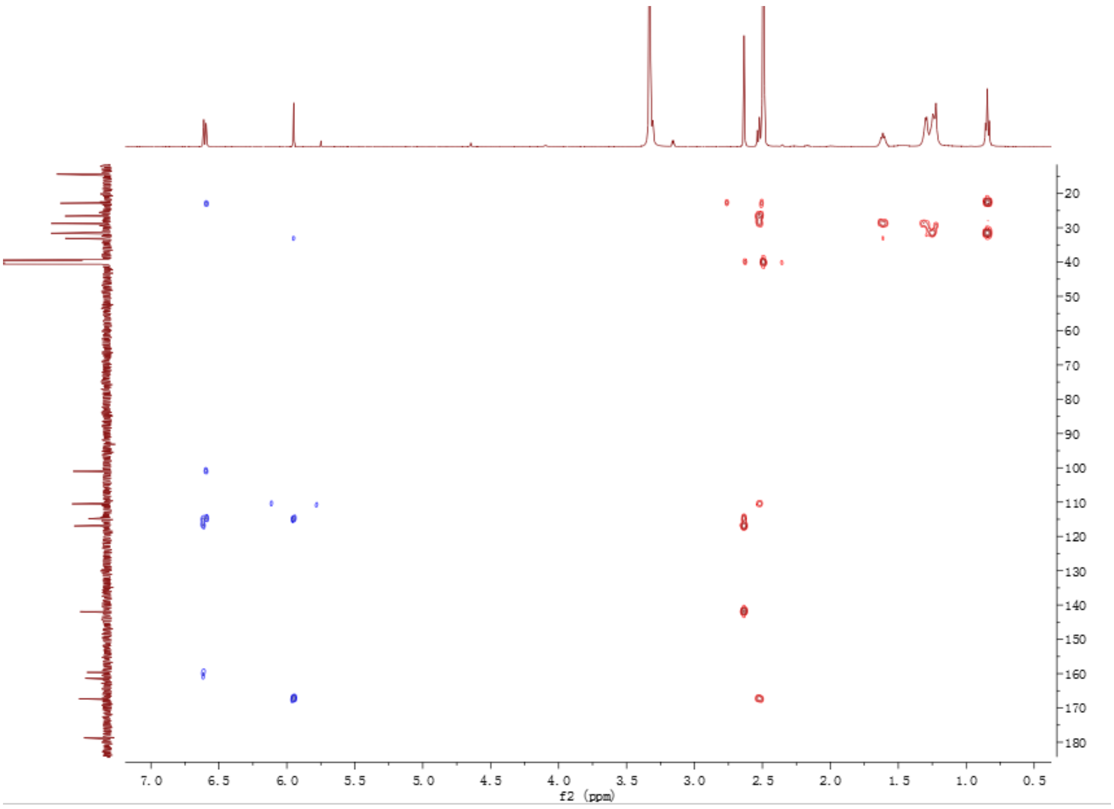

Figure. S35 HMBC spectrum of compound **13**.

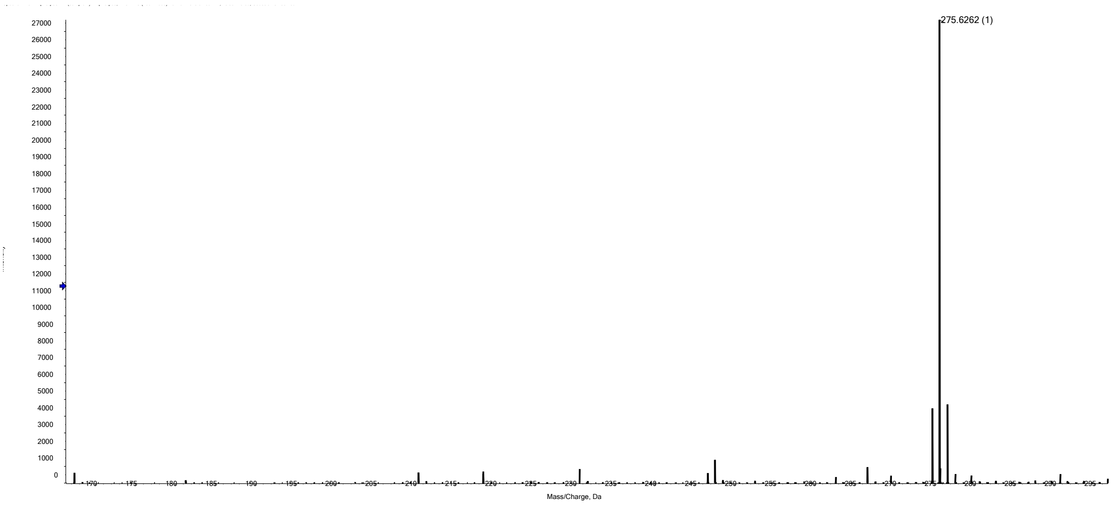

Figure. S36 HRESIMS spectrum of compound **13**.

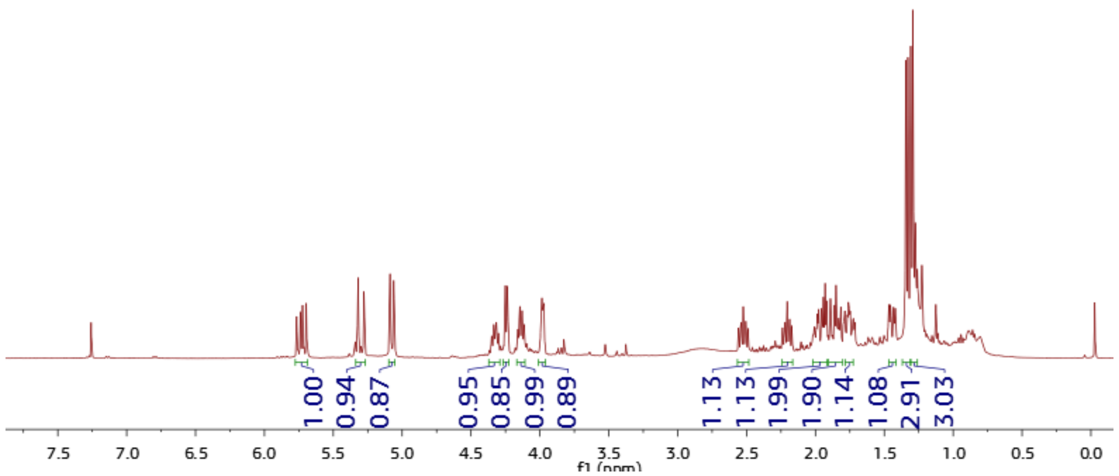

Figure. S37 ^{1}H NMR spectrum of compound **14** (500 MHz, DMSO-d_6).

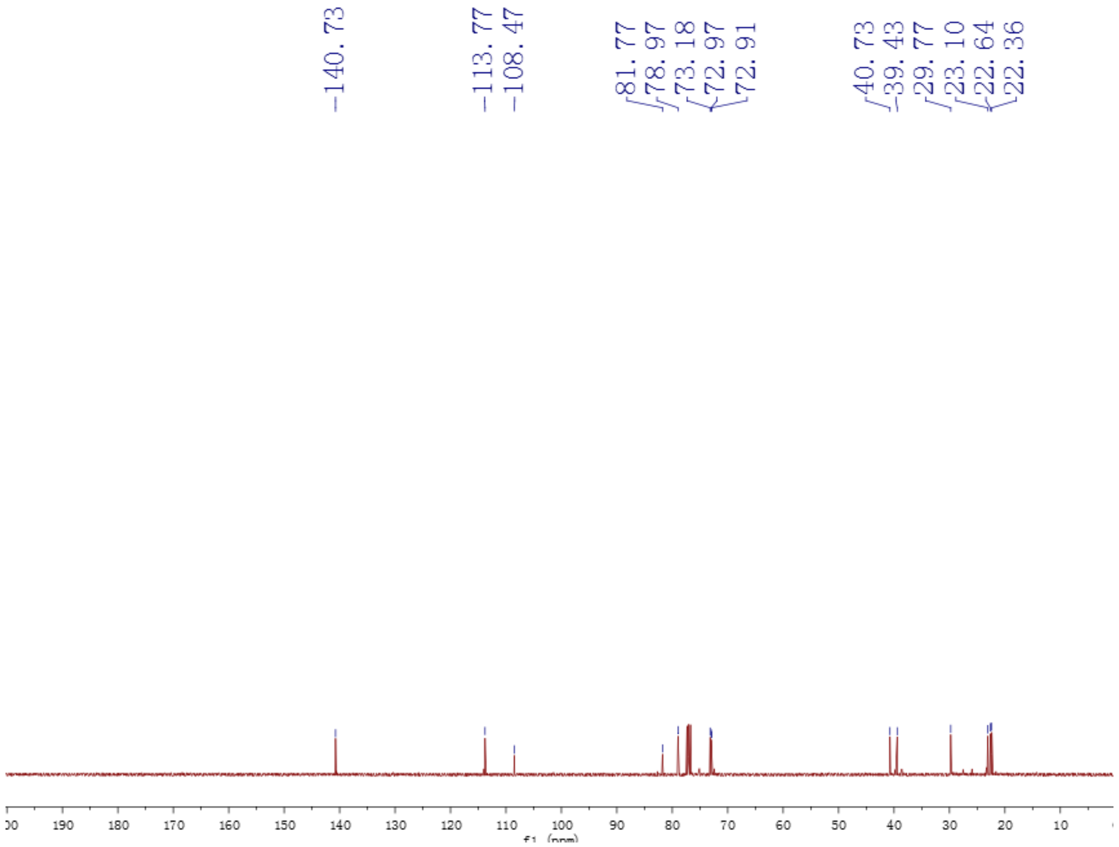

Figure. S38 ^{13}C NMR spectrum of compound **14** (125 MHz, DMSO-d_6).

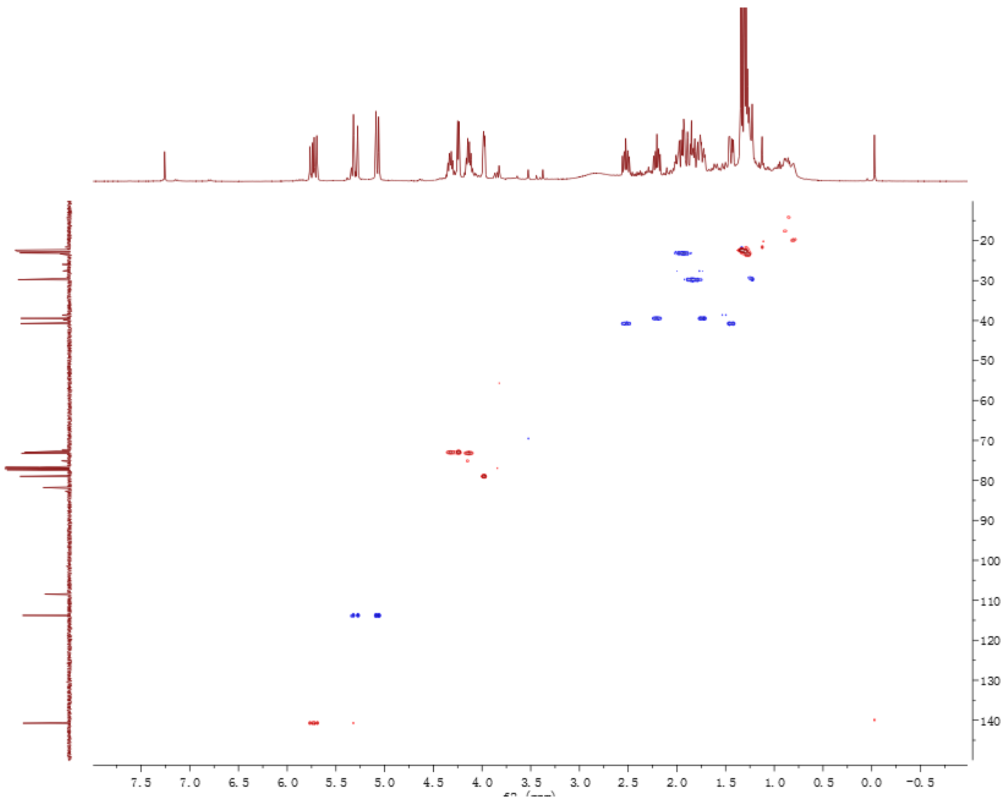

Figure. S39 HSQC spectrum of compound **14**.

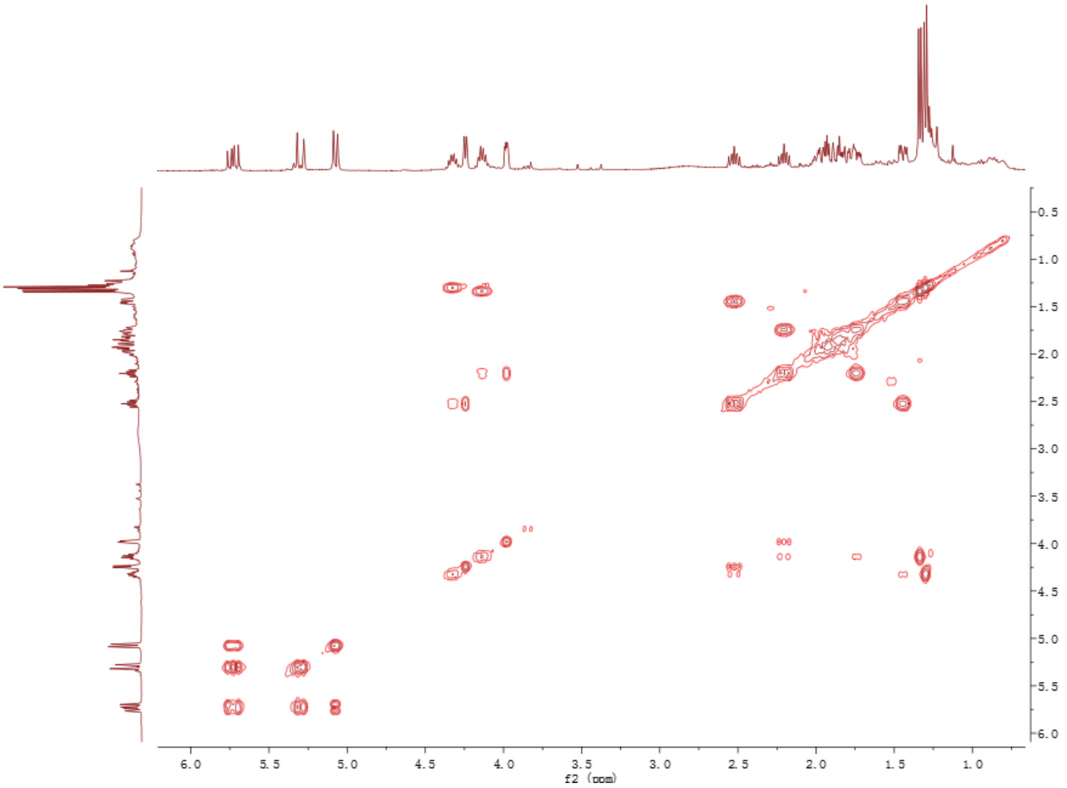

Figure. S40 ^{1}H-^{1}H COSY spectrum of compound **14**.

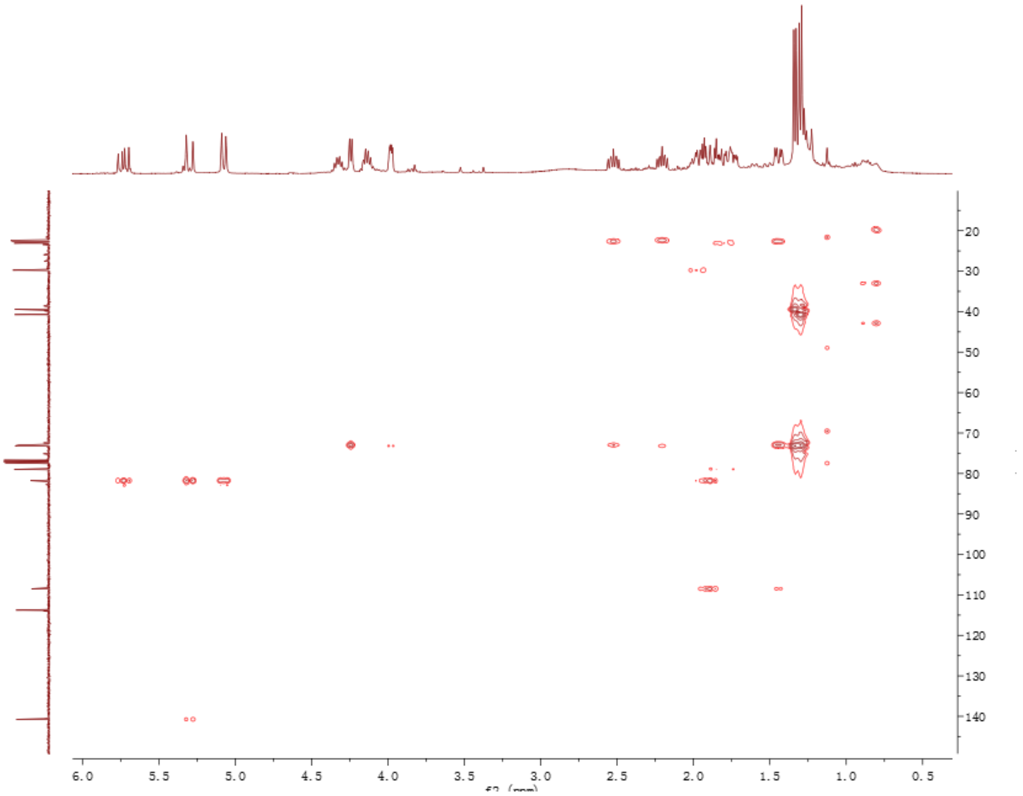

Figure. S41 HMBC spectrum of compound **14**.

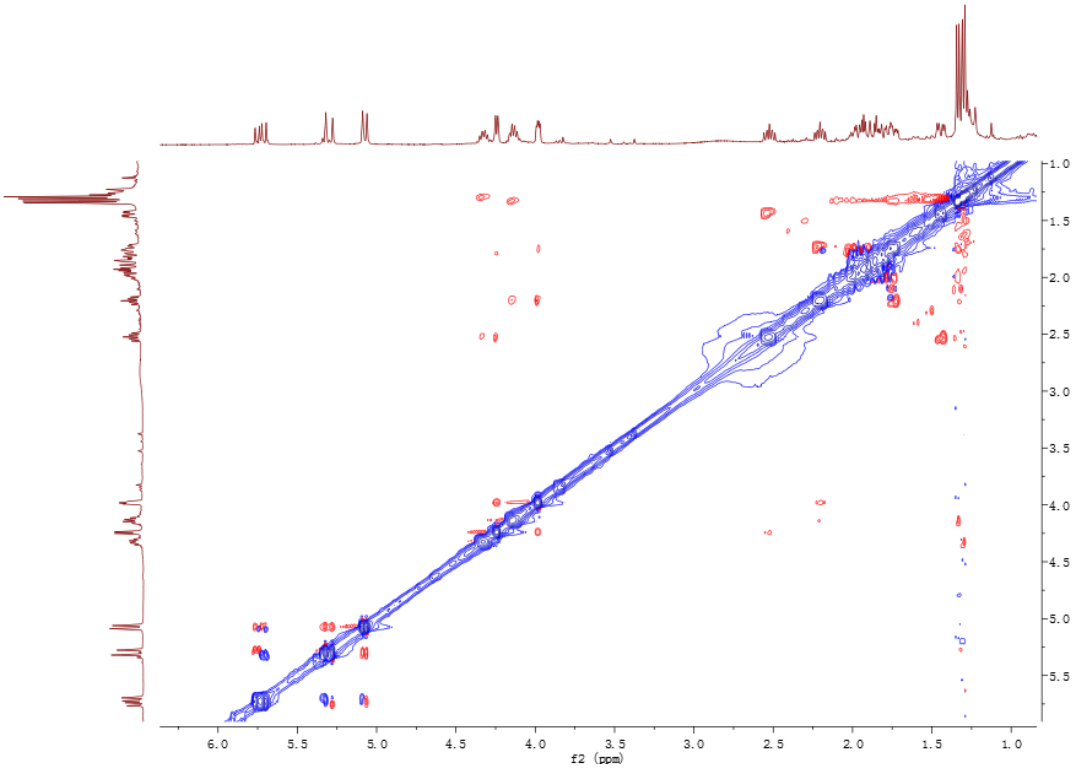

Figure. S42 NOESY spectrum of compound **14**.

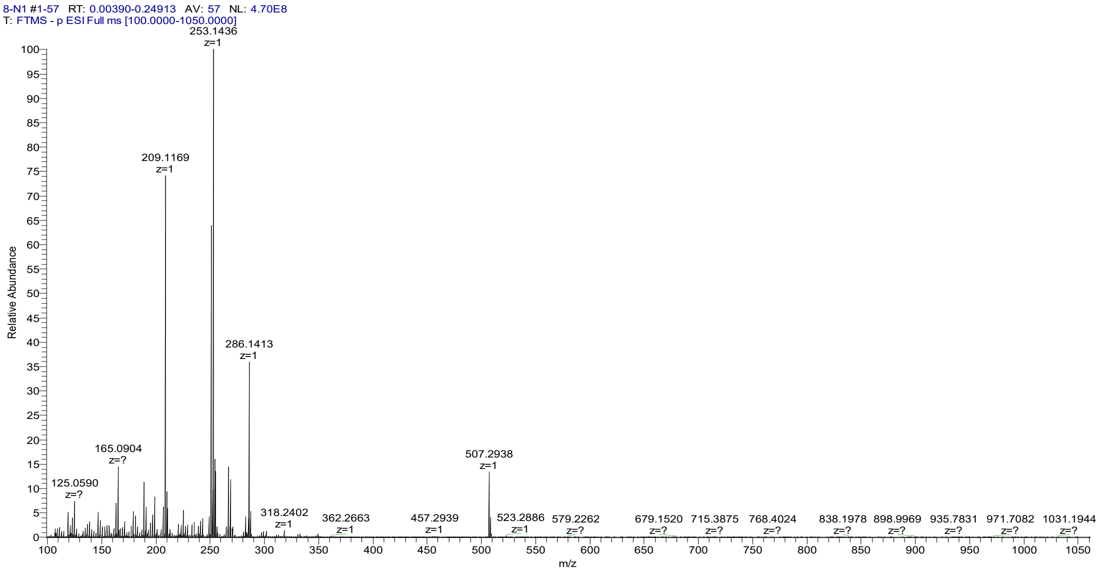

Figure. S43 HRESIMS spectrum of compound **14**.

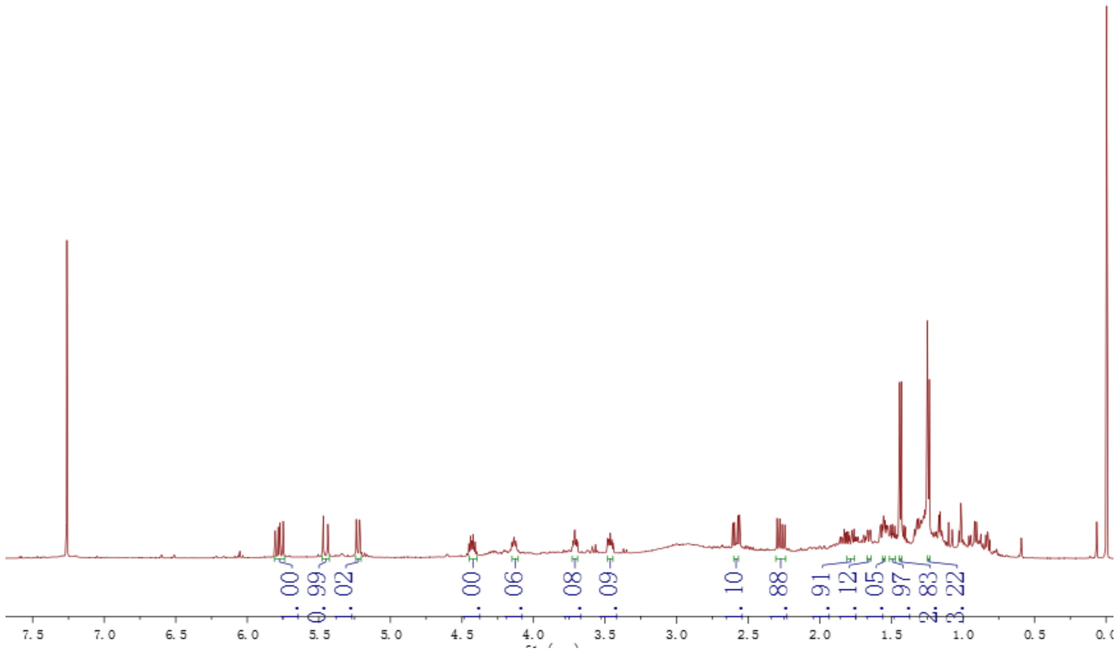

Figure. S44 ^{1}H NMR spectrum of compound **15** (500 MHz, DMSO-d_6).

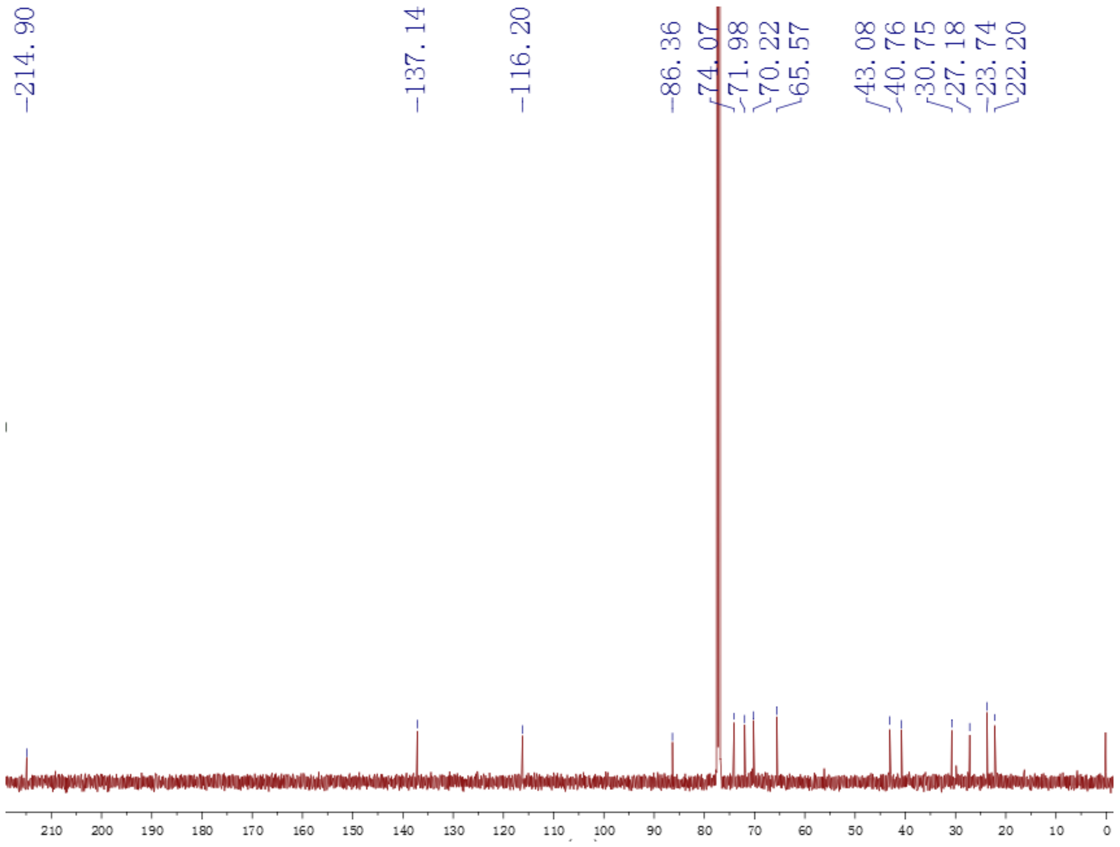

Figure. S45 ^{13}C NMR spectrum of compound **15** (125 MHz, DMSO-d_6).

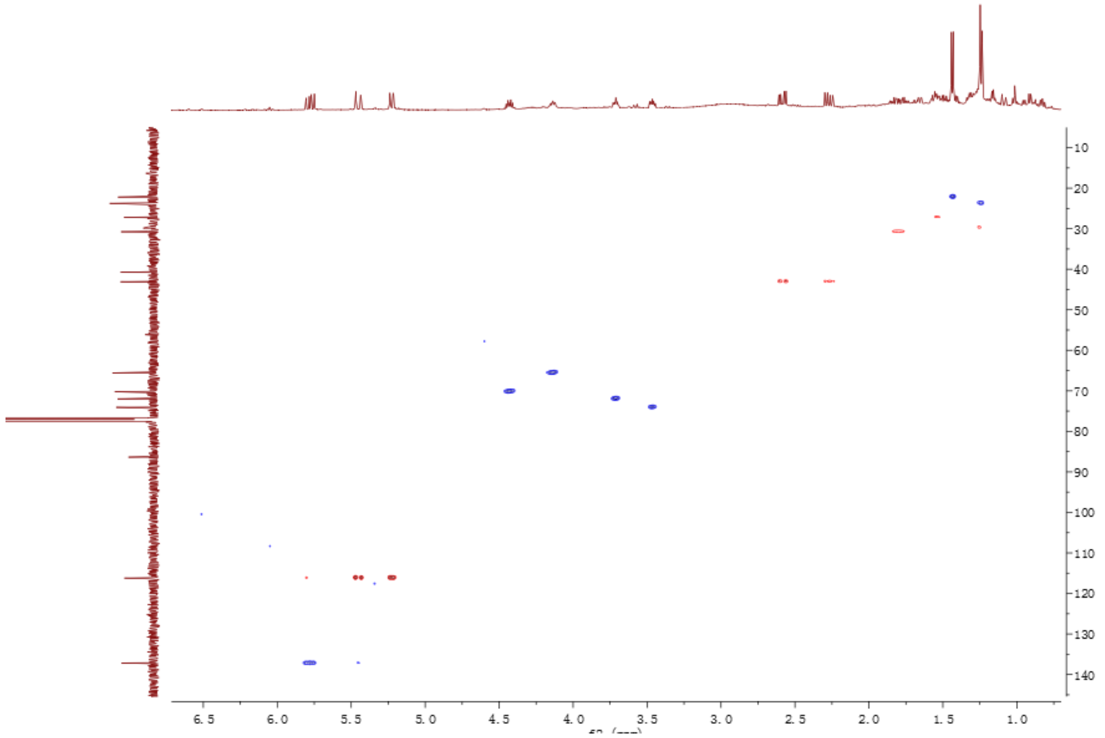

Figure. S46 HSQC spectrum of compound **15**.

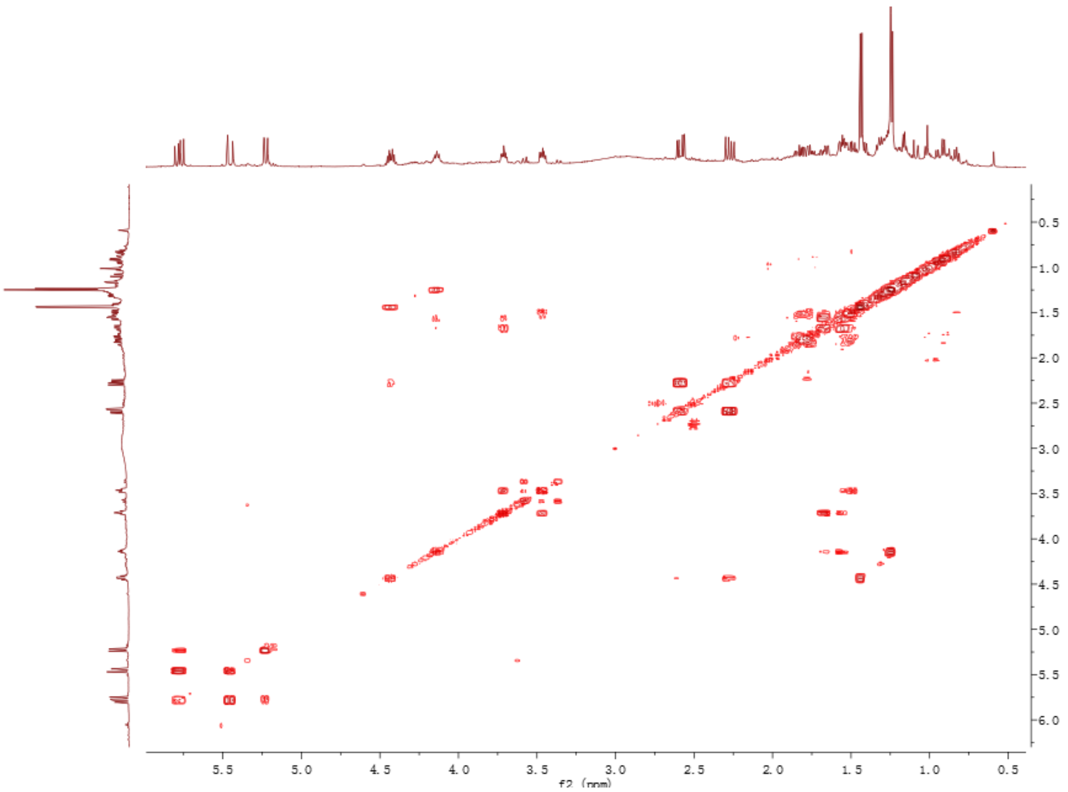

Figure. S47 ^{1}H-^{1}H COSY spectrum of compound **15**.

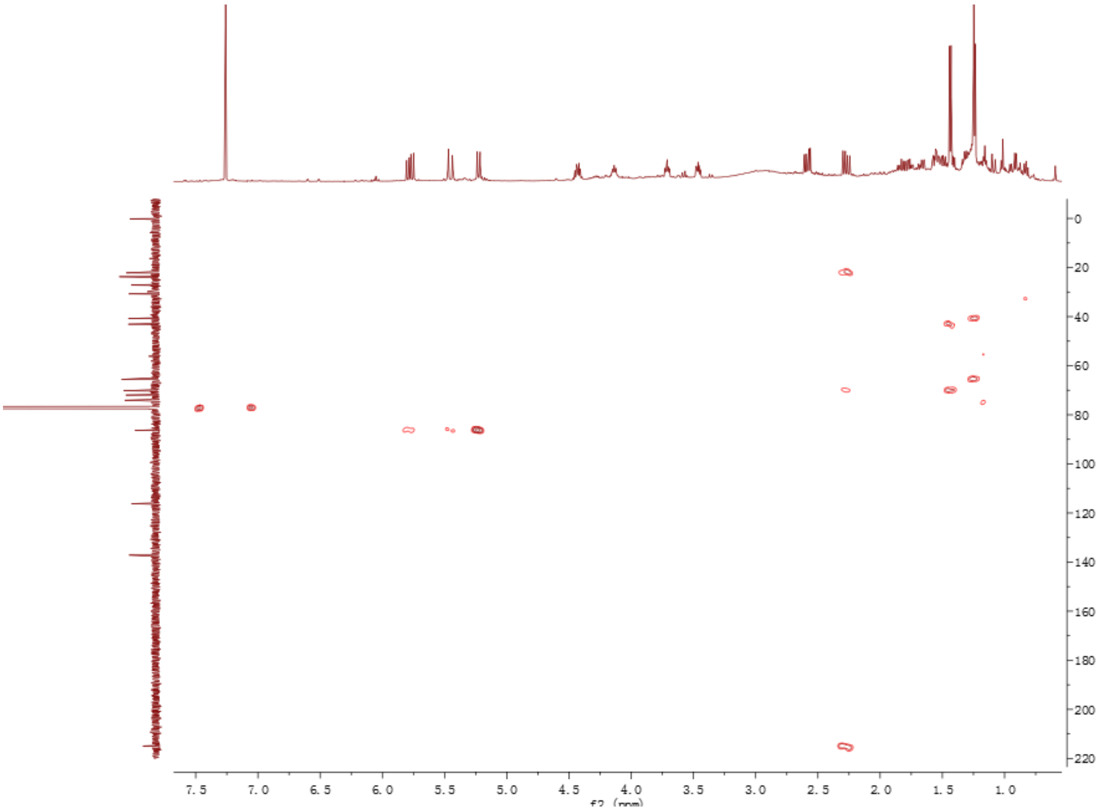

Figure. S48 HMBC spectrum of compound **15**.

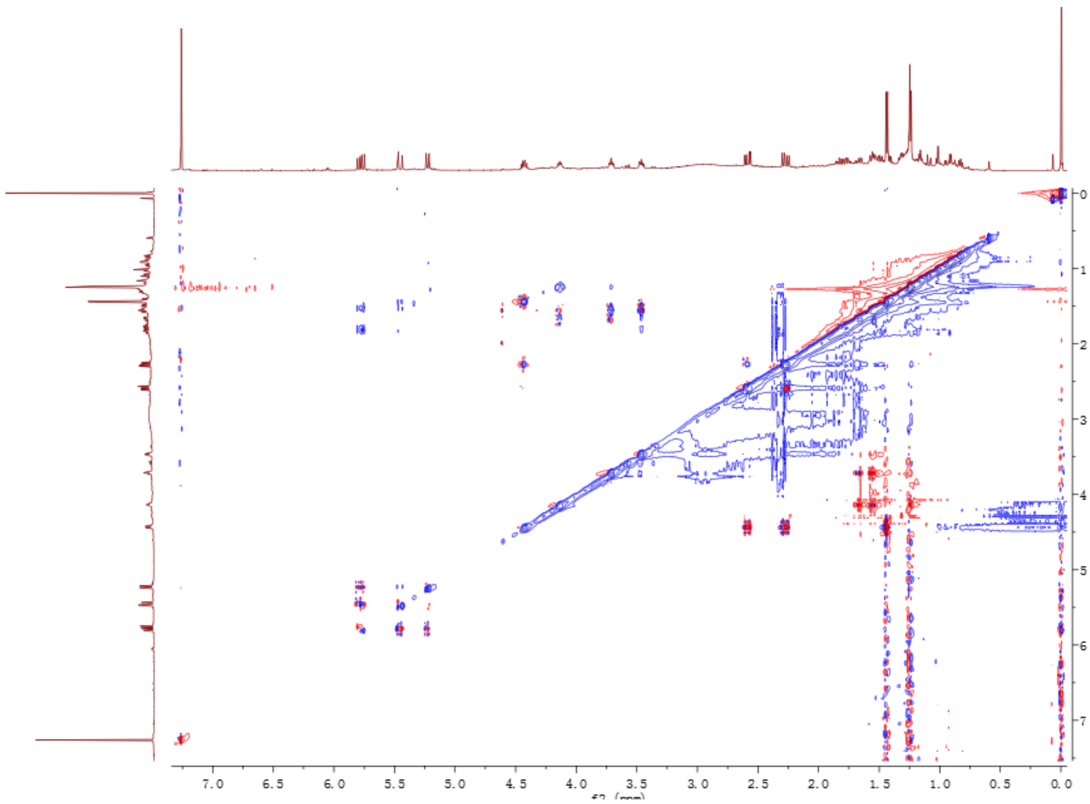

Figure. S49 NOESY spectrum of compound **15**.

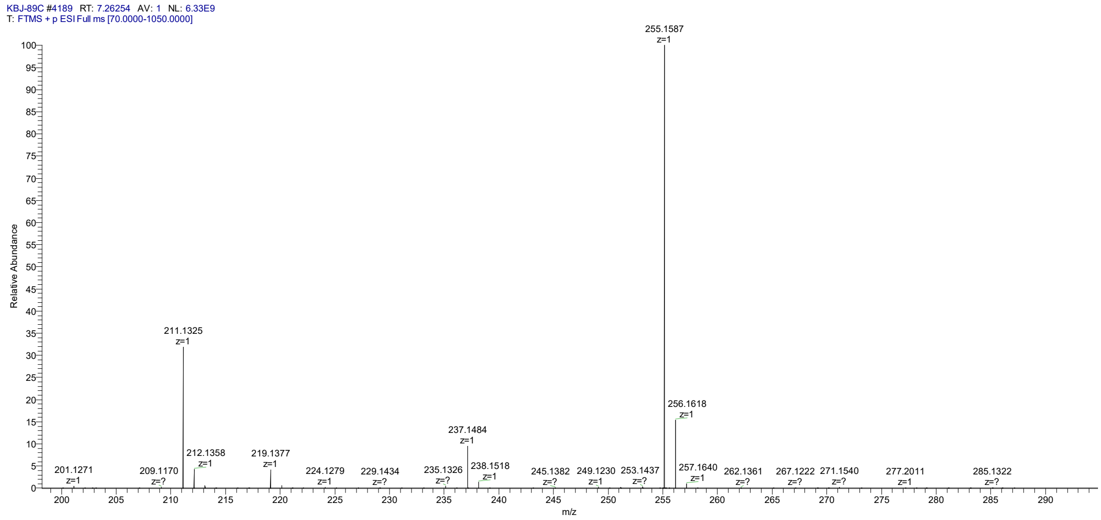

Figure. S50 HRESIMS spectrum of compound **15**.

Table S1. The DP4+ evaluation of compound **14**.

	A	B	C	D	E	F	G	H
1	Functional		Solvent?		Basis Set		Type of Data	
2	mPW1PW91		PCM		6-311+G(d,p)		Unscaled Shifts	
3								
12			DP4+	0.00%	100.00%	-	-	-
14	Nuclei	sp2?	xperimenta	Isomer 1	Isomer 2	Isomer 3	Isomer 4	Isomer 5
15	C		73	72.5	74.8			
16	C		40.7	40.8	39.1			
17	C		72.9	80.5	73.0			
18	C		81.8	78.2	83.9			
19	C		23.1	28.9	24.5			
20	C		29.8	31.5	29.5			
21	C		108.5	102.6	107.8			
22	C		79	77.1	80.3			
23	C		39.4	40.0	41.1			
24	C		73.2	73.9	73.8			
25	C		22.4	21.7	20.2			
26	C	x	140.7	144.30	140.90			
27	C	x	113.8	111.70	113.70			
28	C		22.6	20.70	21.50			
29	H		4.33	3.94	4.18			
30	H		2.52	2.53	2.31			
31	H		1.44	2.13	1.49			
32	H		4.24	4.34	4.08			
33	H		1.85	1.89	1.86			
34	H		1.94	1.69	1.71			
35	H		3.98	3.87	3.98			
36	H		2.21	2.19	2.53			
37	H		1.34	1.65	1.36			
38	H		4.14	4.02	4.08			
39	H		1.34	1.28	1.29			
40	H	x	5.73	6.33	5.96			
41	H	x	5.3	5.39	5.38			
42	H	x	5.07	5.2	5.19			
43	H		1.3	1.25	1.25			

	A	B	C	D	E	F	G	H
1	Functional		Solvent?		Basis Set		Type of Data	
2	mPW1PW91		PCM		6-311+G(d,p)		Unscaled Shifts	
3								
4			Isomer 1	Isomer 2	Isomer 3	Isomer 4	Isomer 5	Isomer 6
5	sDP4+ (H data)		0.03%	99.97%	-	-	-	-
6	sDP4+ (C data)		0.00%	100.00%	-	-	-	-
7	sDP4+ (all data)		0.00%	100.00%	-	-	-	-
8	uDP4+ (H data)		0.32%	99.68%	-	-	-	-
9	uDP4+ (C data)		0.01%	99.99%	-	-	-	-
10	uDP4+ (all data)		0.00%	100.00%	-	-	-	-
11	DP4+ (H data)		0.00%	100.00%	-	-	-	-
12	DP4+ (C data)		0.00%	100.00%	-	-	-	-
13	DP4+ (all data)		0.00%	100.00%	-	-	-	-

Table S2. The binding energy of compounds **1-13** with numerous inflammatory targets (kcal/mol).

PDB ID	protein name	compounds												
		**1**	**2**	**3**	**4**	**5**	**6**	**7**	**8**	**9**	**10**	**11**	**12**	**13**
2ORP	iNOS	-11.1	-7.7	-7.8	-9.2	-10.8	-11.0	-7.3	-11.0	-10.6	-9.5	-9.0	-10.9	-10.4
1HT8	COX-1	-10.5	-6.6	-8.6	-3.5	-6.3	-7.6	-6.7	-10.7	-7.3	-5.5	-6.6	-9.3	-8.5
5F19	COX-2	-10.1	-6.9	-8.3	-2.7	-8.3	-11.1	-11.2	-11.7	-11.3	-11.3	-10.8	-12.3	-8.6
6S8T	ICAM	-8.3	-7.1	-9.6	-6.0	-7.3	-10.8	-9.3	-7.2	-8.5	-8.2	-8.5	-9.7	-7.6
4HSA	IL-17	-8.5	-8.6	-7.6	-8.8	-8.1	-11.0	-8.1	-9.5	-10.0	-8.9	-10.5	-0.66	-9.9
3QT2	IL-5	-10.3	-7.6	-8.9	-8.4	-7.7	-8.6	-8.2	-6.8	-7.7	-8.1	-9.4	-8.2	-6.3
6N7A	JAK1	-7.2	-9.2	-8.9	-6.7	-8.7	-9.2	-9.5	-9.5	-10.9	-8.7	-7.0	-8.4	-7.8

6X8E	JAK2	-8.0	-7.1	-7.3	-7.6	-7.1	-9.7	-9.9	-10.8	-9.6	-7.2	-8.3	-8.8	-6.8
4RMH	SIRT2	--8.1	-6.5	-8.5	-5.7	-8.0	-6.1	-7.3	-9.2	-8.8	-7.6	-9.3	-5.3	-9.9
2AZ5	TNF-α	-8.3	-6.9	-8.4	-7.2	-7.1	-10.2	-6.4	-9.2	-8.8	-7.0	-9.6	-7.8	-7.5
